# No Evidence of a Common DNA Variant Profile Specific to World Class Endurance Athletes

**DOI:** 10.1371/journal.pone.0147330

**Published:** 2016-01-29

**Authors:** Tuomo Rankinen, Noriyuki Fuku, Bernd Wolfarth, Guan Wang, Mark A. Sarzynski, Dmitry G. Alexeev, Ildus I. Ahmetov, Marcel R. Boulay, Pawel Cieszczyk, Nir Eynon, Maxim L. Filipenko, Fleur C. Garton, Edward V. Generozov, Vadim M. Govorun, Peter J. Houweling, Takashi Kawahara, Elena S. Kostryukova, Nickolay A. Kulemin, Andrey K. Larin, Agnieszka Maciejewska-Karłowska, Motohiko Miyachi, Carlos A. Muniesa, Haruka Murakami, Elena A. Ospanova, Sandosh Padmanabhan, Alexander V. Pavlenko, Olga N. Pyankova, Catalina Santiago, Marek Sawczuk, Robert A. Scott, Vladimir V. Uyba, Thomas Yvert, Louis Perusse, Sujoy Ghosh, Rainer Rauramaa, Kathryn N. North, Alejandro Lucia, Yannis Pitsiladis, Claude Bouchard

**Affiliations:** 1 Human Genomics Laboratory, Pennington Biomedical Research Center, Louisiana State University System, Baton Rouge, Louisiana, United States of America; 2 Graduate School of Health and Sports Science, Juntendo University, Chiba, Japan; 3 Department of Sport Medicine Humboldt University and Charite University School of Medicine, Berlin, Germany; 4 Centre for Sport and Exercise Science and Medicine (SESAME), University of Brighton, Eastbourne, United Kingdom; 5 Research Institute for Physical-Chemical Medicine, Moscow, Russia; 6 Sport Technology Research Centre, Volga Region State Academy of Physical Culture, Sport and Tourism, Kazan, Russia; 7 Department of Kinesiology, Laval University, Ste-Foy, Québec, Canada; 8 University of Szczecin, Department of Physical Education and Health Promotion, Szczecin, Poland; 9 Academy of Physical Education and Sport, Department of Tourism and Recreation, Gdansk, Poland; 10 Institute of Sport, Exercise and Active Living (ISEAL), Victoria University, Victoria, Australia; 11 Pharmacogenomics Laboratory, Institute of Chemical Biology and Fundamental Medicine of SB RAS, Novosibirsk, Russia; 12 Novosibirsk State University, Novosibirsk, Russia; 13 Murdoch Childrens Research Institute and Department of Paediatrics, University of Melbourne, Victoria, Australia; 14 Institute of Neuroscience and Muscle Research, Childrens Hospital Westmead, Westmead, Australia; 15 Department of Sports Medicine, Japan Institute of Sports Sciences, Tokyo, Japan; 16 Department of Health Promotion and Exercise, National Institute of Health and Nutrition, Tokyo, Japan; 17 Universidad Europea and Research Institute i+12, Madrid, Spain; 18 College of Medicine, Veterinary & Life Sciences, Institute of Cardiovascular & Medical Sciences, University of Glasgow, Glasgow, United Kingdom; 19 MRC Epidemiology Unit, University of Cambridge School of Clinical Medicine, Institute of Metabolic Science, Cambridge Biomedical Campus, Cambridge, United Kingdom; 20 Federal Medical Biological Agency, Moscow, Russia; 21 Cardiovascular & Metabolic Disorders Program, and Center for Computational Biology, Duke-NUS Graduate Medical School, Singapore, Singapore; 22 Kuopio Research Institute of Exercise Medicine, University of Eastern Finland, Kuopio, Finland; 23 School of Public Health, University of South Carolina, Columbia, SC, United States of America; University of Northampton, UNITED KINGDOM

## Abstract

There are strong genetic components to cardiorespiratory fitness and its response to exercise training. It would be useful to understand the differences in the genomic profile of highly trained endurance athletes of world class caliber and sedentary controls. An international consortium (GAMES) was established in order to compare elite endurance athletes and ethnicity-matched controls in a case-control study design. Genome-wide association studies were undertaken on two cohorts of elite endurance athletes and controls (GENATHLETE and Japanese endurance runners), from which a panel of 45 promising markers was identified. These markers were tested for replication in seven additional cohorts of endurance athletes and controls: from Australia, Ethiopia, Japan, Kenya, Poland, Russia and Spain. The study is based on a total of 1520 endurance athletes (835 who took part in endurance events in World Championships and/or Olympic Games) and 2760 controls. We hypothesized that world-class athletes are likely to be characterized by an even higher concentration of endurance performance alleles and we performed separate analyses on this subsample. The meta-analysis of all available studies revealed one statistically significant marker (rs558129 at *GALNTL6* locus, p = 0.0002), even after correcting for multiple testing. As shown by the low heterogeneity index (I^2^ = 0), all eight cohorts showed the same direction of association with rs558129, even though p-values varied across the individual studies. In summary, this study did not identify a panel of genomic variants common to these elite endurance athlete groups. Since GAMES was underpowered to identify alleles with small effect sizes, some of the suggestive leads identified should be explored in expanded comparisons of world-class endurance athletes and sedentary controls and in tightly controlled exercise training studies. Such studies have the potential to illuminate the biology not only of world class endurance performance but also of compromised cardiac functions and cardiometabolic diseases.

## Introduction

Early studies on the genetic basis of sports performance reported that identical twins who engaged in competitive sports were significantly more likely to participate in the same sports than pairs of dizygotic twins [1]. The first documented attempts to identify genetic markers for sports performance date to the 1968 Mexico and 1976 Montreal Olympic Games and were based on common blood genetic markers. They did not yield any strong positive findings [2–4]. A high maximal oxygen uptake (VO_2_max) is a necessary condition to reach the level of an endurance athlete of international caliber. A high VO_2_max can only be achieved if an individual is endowed with a very high level in the sedentary state (intrinsic level) in combination with large increases in response to sustained and demanding exercise training regimens (trainability).

Twin and family studies have revealed that the intrinsic level of VO_2_max is strongly influenced by a genetic component. For instance, the heritability of VO_2_max adjusted for age, sex and body composition in sedentary families of European descent reached 51% in the HERITAGE Family Study [5]. Evidence of a significant heritability level has also been documented for exercise training-induced improvements in VO_2_max. Large individual differences in VO_2_max gains have been found in sedentary young adults subjected to standardized endurance training programs [6]. A series of exercise training experiments conducted with pairs of identical twins revealed that the differences in trainability were not distributed randomly among the twins, with intraclass correlations in response of VO_2_max (L O_2_/min) ranging from 0.44 to 0.77 [7–9], indicating that members of the same twin pair responded similarly to training. In HERITAGE, the increase in VO_2_max in 481 individuals from 99 two-generation families of Whites of European descent showed 2.5 times more variance between families than within families for VO_2_max response, with a maximal heritability estimate of 47% [10]. Adjusting the VO_2_max response data for baseline VO_2_max did not modify this estimate, suggesting along with other evidence that the familial and genetic factors underlying VO_2_max in the sedentary state and its response to exercise training are different [11].

In the case-control GENATHLETE study, single nucleotide polymorphisms (SNPs) in several candidate genes were investigated but none have provided strong evidence for differences in allele and genotype frequencies between elite endurance athletes and controls [12–17]. Reports published to date have focused on differences in allele frequencies between athletes and non-athlete controls mainly on angiotensin I converting enzyme (*ACE)*, α-actinin-3 (*ACTN3)*, and peroxisome proliferator-activated receptor-γ coactivator 1α (*PPARGC1A)* polymorphisms and on mitochondrial DNA (mtDNA) haplogroup distributions [18]. There were several positive findings but most were derived from *post hoc* subgroup analyses and only a few studies controlled for multiple testing. Most of prior studies have come from elite Japanese runners [19, 20], Spanish endurance athletes [21], Ethiopian and Kenyan endurance runners [22, 23], Russian endurance athletes [24] as well as endurance athletes from Australia [25], Israel [26] and Poland [27].

Unbiased genome-wide approaches have been used in the search for genomic regions, transcripts or DNA variants linked or associated with endurance performance-related traits [28–31]. For instance, in a recent study, the association of 324,611 SNPs with the response of VO_2_max to endurance training in 473 Whites from HERITAGE was investigated [28]. None of the SNPs reached genome-wide significance even though there were several SNPs moderately associated with VO_2_max trainability.

It is of scientific interest to understand the differences in the genomic profile of highly trained endurance athletes of world class caliber and sedentary controls from the same ethnic ancestry. Such data could illuminate the biology of cardiorespiratory fitness and human adaptability and would likely have implications for common health problems such as those observed in aging individuals with declining health, diabetic patients with compromised cardiovascular fitness or patients with ischemic heart disease or heart failure to name but a few. Given the heterogeneous results reported in previous studies, we established an international consortium (GAMES) in order to reach larger sample sizes of elite endurance athletes and matched controls in a case-control study design. Genome-wide explorations were undertaken on two cohorts of world-class endurance athletes and controls (GENATHLETE and Japanese endurance runners), which generated a panel of 45 markers that were subsequently used for replication in seven additional cohorts of endurance athletes and controls.

## Participants and Methods

The GENATHLETE cohort and a sample of endurance athletes and controls from Japan were used for the discovery phase. The characteristics of the GENATHLETE all male participants are presented in S1 Table and S1 Fig of the Supporting Information. There were 315 elite endurance athletes (national and world-class level) from Germany, Finland, Canada and the USA plus 320 sedentary controls from the same countries. All GENATHLETE participants are males and athletes and controls were closely matched for ethnicity and country of origin. The mean highest recorded VO_2_max of these athletes was 79.0 mL O_2_/kg/min with a standard deviation (SD) of 3.4 mL O_2_ while the mean value for the sedentary controls was 40.0 (SD = 7.1). In the Japanese sample, 60 Japanese elite runners (world-class athletes) and 116 controls were available for the discovery phase. (Additional information on these two cohorts is provided in Supporting Information).

Endurance athletes and controls from 7 countries were used for the replication phase (Table 1). Participants were from Australia, Ethiopia, Japan, Kenya, Poland, Russia and Spain. It should be noted that we used a second Japanese cohort (143 athletes, 692 controls) in the replication phase. Again, in each country, endurance athletes were competing at least at the national level and most of them competed in Olympic or world cup events. A total of 1520 athletes and 2760 controls were involved in the present study. Among the endurance athletes, 1045 (about 69% of total) took part in endurance events at world championships and Olympic Games. We hypothesized that these world-class athletes are likely to be characterized by a higher concentration of “endurance performance alleles” and we performed separate analyses on this subsample (see Supporting Information for details).

**Table 1:** Number of Athletes and Controls of Each Study Participating in the GAMES Consortium.

Study	Number of Athletes	Number of World-Class Athletes*	Number of Controls
**DISCOVERY PHASE**			
GENATHLETE Study	315	168	320
(Germany, Finland, Canada, USA)			
Japan Study	60	60	116
**REPLICATION PHASE**			
Australia Study	207–215	160–167	252–258
Ethiopia Study	74–75	74–75	196–198
Japan	137–143		688–692
Kenya Study	269–276	66	79–83
Poland Study	98–113	50–61	136–161
Russia Study	130–153	63–68	187–734
Spain Study	165–170	165–170	184–198
**TOTAL**	**1520**	**835**	**2760**

* World-Class athletes were defined as either having competed at the world championship level and/or in Olympic Games or, in the case of GENATHLETE, as having a maximal oxygen uptake (VO_2_max) > 78 ml O_2_/kg/min. The range reflects the smallest and largest numbers of participants available for any given SNP.

### Discovery Phase

GAMES was established after the two studies (GENATHLETE and Japanese endurance athlete cohort) had already performed their genome-wide screen and performed their analyses. Thus there are differences in the chips used and the analytical strategies. However, the data of the replication studies were all analyzed centrally at the Pennington site.

In GENATHLETE, genomic DNA was extracted from whole-blood samples by commercial DNA extraction kits (Gentra Systems, Inc., Minneapolis, MN), and the DNA stock samples were diluted to 50 ng/μl concentrations. SNPs for the study were those captured in the Illumina CardioMetabochip (Illumina Inc., San Diego, CA), which contains over 195,000 genetic markers including ~66,000 variants implicated in the aetiology of cardiometabolic traits and disease outcomes from discovery GWAS cohorts, as well as variants around known loci for the purposes of fine-mapping [32]. The SNPs were genotyped using the Illumina Infinium II assay on Illumina iScan platform.

SNPs showing marked deviation from Hardy-Weinberg equilibrium (HWE) (p ≤ 0.00001) were excluded. However, since deviations from HWE may be related to case-control status-related differences of genotype frequencies, identical non-HWE pattern was confirmed both in endurance athletes and non-athlete controls before the SNP was excluded from the database. A total of 143,000 SNPs were polymorphic and passed the quality control filters. In GENATHLETE, we estimate that we an 80% statistical power to detect odds ratios (ORs) of 2.7 and 2.1 for minor allele frequencies of 0.1 and 0.3, respectively, assuming an additive model and an alpha level of 5x10^-8^.

For the Japanese cohort, total DNA was isolated from saliva or venous blood by use of QIAamp DNA blood Maxi Kit (QIAGEN, Hilden, Germany) or Oragene DNA Collection Kits (DNA genotek, Ontario, Canada), respectively. Total DNA samples were genotyped for more than 700,000 markers using the Illumina^®^ HumanOmniExpress Beadchip. The genotype calls were performed with the Illumina GenomeStudio software. Quality control measures were performed as defined in the Supporting Information. After removing SNPs failing quality control, 541,179 autosomal SNPs in 60 Japanese endurance athletes and 116 Japanese controls were available for association analyses. In the Japanese cohort, we have the ability to detect at 80% statistical power ORs of 6.1 and 4.5 for minor allele frequencies of 0.1 and 0.3, respectively, assuming an additive model and an alpha level of 5x10^-8^.

### Data Analysis

For the GENATHLETE samples, tests of HWE for each SNP were conducted using the exact test implemented in the PEDSTATS software [33]. Allele frequency differences between athletes and controls were tested using a chi-square test as implemented in the PLINK software package [34]. For the Japanese study, standard allelic association analysis was performed by comparing allele-frequency differences between Japanese endurance runners and controls.

None of the SNPs reached genome-wide significance level (p<5 x 10^−8^) in GENATHLETE or the Japanese Study. Considering that this may be simply the result of the relatively small sample size of the discovery cohorts, we elected to retain the 45 most promising SNPs (all p<1 x 10^−4^) for further testing in the replication studies. Among these 45 SNPs, 26 came from GENATHLETE CardioMetabochip (13 based on full cohort, 13 from the highest VO_2_max subgroup analyses) and 19 from the Japanese GWAS.

### Replication Phase

The 45 SNPs carried forward were genotyped in athletes and controls from Australia, Ethiopia, Japan, Kenya, Poland, Russia and Spain as described in the Supporting Information. Tests of HWE for each SNP were also performed with the PEDSTATS software [33] and allele-frequency differences between athletes and controls were tested using a chi-square test as implemented in PLINK [34].

### Meta-analysis

Results from individual studies were combined using a meta-analysis approach. These analyses were done with the meta-analysis routine of the PLINK software using study-specific association test result files as an input. Three subsets were meta-analyzed: all studies based on athletes of European descent that were part of the replication phase (i.e. Australia, Poland, Russia and Spain), the two studies based on participants from Africa, and all studies combined (including athletes from Ethiopia, Kenya and Japan). In each case, analyses were undertaken based on the results of the comparisons between all athletes and controls from each national cohort.

### Further Analyses Performed on World-Class Endurance Athletes

In some of the national cohorts, all endurance athletes were of world-class caliber. However, in other cohorts (GENATHLETE, Poland, Russia and the second Japanese cohort), there was a combination of national level and world-class caliber endurance athletes. We were able to classify the endurance athletes between those who were truly of world-class caliber, as defined by their participation in World Championship competitions or in Olympic Games, and those who competed at the national level (see Supporting Information). In the case of GENATHLETE, the classification was based on the VO_2_max measurement, with those exhibiting a VO_2_max value ≥78 mL O_2_/kg/min being classified as part of the “super elite” contingent. All the analyses described in the previous sections were repeated using only these world-class super elite endurance athletes and controls.

### Informed Consent

The GENATHLETE project was originally approved by the Medical Ethics Committee of Laval University (Quebec, Canada). Approval has also been obtained from the medical ethics committees of all institutions that have contributed participantsto the GENATHLETE cohort. Continuing approval for the study has been granted by the IRB of Pennington Biomedical Research Center. Each GENATHLETE participant has given written informed consent. Written informed consent was obtained from all participants from the Japanese, Ethiopian, and Kenyan cohorts, respectively, and was approved by the Institutional Review Board of Tokyo Metropolitan Institute of Gerontology, National Institute of Health and Nutrition, Japan; the Oxford Tropical Research Ethics Committee, the University of Glasgow Ethics Committee, and a committee from the Ethiopian Athletics Federation; and the University Ethics Committee in Kenya. Written informed consent was obtained for all Australian participants. The study was approved by the institutional review boards and Ethics Committees of the Children’s Hospital at Westmead, the University of Sydney, and the Australian Institute of Sport. The procedures followed in the study from Poland were approved by the Pomeranian Medical University Ethics Committee and all participants gave informed written consent. The elite athlete study from Russia was approved by the Ethics Committee of the Research Institute for Physical-Chemical Medicine. Written informed consent was obtained from each participant. All participants from the Spanish cohort provided written consent and the study protocol was approved by the IRB of *Pablo Olavide* University (Spain).

## Results

### Discovery Phase

**Fig 1:**
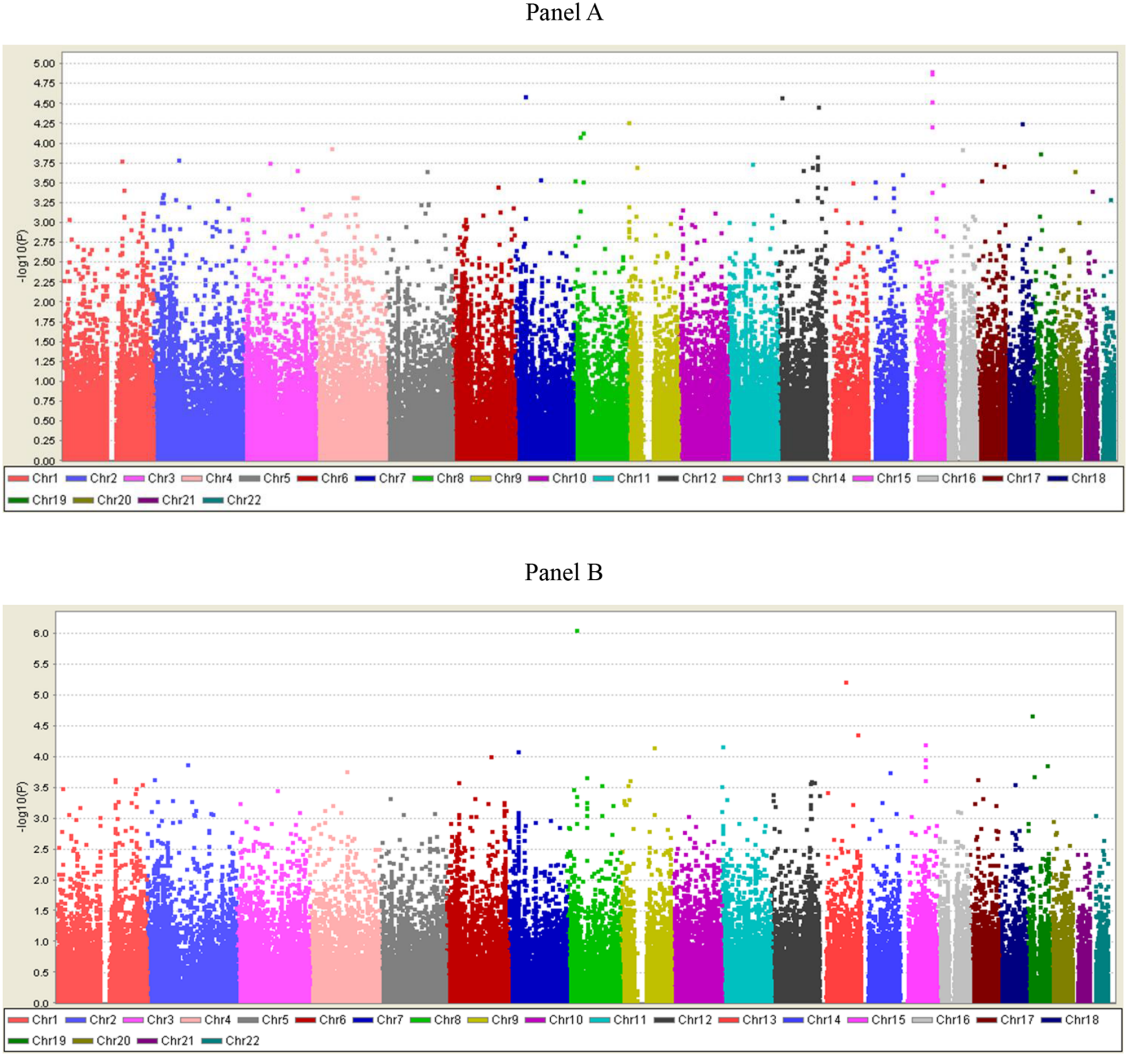
Manhattan Plot of the Association P Values for the CardioMetabochip SNP Differences in Allele Frequencies Between Endurance Athletes and Sedentary Controls in GENATHLETE. (A) Athletes with VO_2_max ≥ 75 ml O_2_/kg/min (N = 315) vs. Controls (N = 320). (B) Elite Athletes with VO_2_max ≥ 78 ml O_2_/kg/min (N = 168) vs. Controls (N = 320).

In GENATHLETE, a total of 143,000 SNPs were polymorphic and passed the quality control filters. A Manhattan plot depicting associations between elite endurance athletes and sedentary controls for all SNPs across the 22 autosomes is shown in Panel A of Fig 1. The strength of the association is shown on the y-axis as a—log10 of the p-value, which represents the statistical significance of the allele frequency chi-square test. None of the SNPs reached the genome-wide significance threshold of 5x10^-8^. 50 SNPs showed p-values of less than 5.0 x 10^−4^ and the top 26 SNPs are shown in Table 2. Among the 50 SNPs, 8 were found to be in strong pairwise linkage disequilibrium (r^2^>0.9) and 2 had a minor allele frequency of <5%. From the 40 SNPs left, the top 26 with the most significant p values in GENATHLETE were retained and are shown in the table.

**Table 2:** Discovery Phase: Most Significant Associations of CardioMetabochip SNPs and All Endurance Athletes as well as World-Class Athletes versus Controls in the GENATHLETE Cohort.

				All Athletes (N = 315)				World-Class Athletes (N = 168)			
SNP	CHR	Gene*	Effect Allele	Freq. in Athletes	Freq. in Controls	OR	P	Freq. in Athletes	Freq. in Controls	OR	P
rs17459307	1	NOS1AP	A	0.043	0.097	0.417	0.00016	0.060	0.097	0.590	0.04566
rs6726044	2	BMP10 (40 kb)	A	0.316	0.222	1.619	0.00016	0.324	0.222	1.684	0.00050
rs2118908	2	ACOXL	G	0.129	0.083	1.634	0.00796	0.164	0.083	2.168	0.00013
rs6799372	3	CNTN3	A	0.189	0.278	0.604	0.00017	0.191	0.278	0.611	0.00259
rs10938202	4	ATP8A1	C	0.113	0.053	2.264	0.00012	0.110	0.053	2.206	0.00113
rs4699824	4	PPP3CA (222 kb)	G	0.402	0.308	1.509	0.0004771	0.429	0.308	1.687	0.00017
rs10499127	6	NKAIN2	G	0.129	0.069	1.999	0.00035	0.146	0.069	2.313	9.74x10^-5^
rs17055965	8	ADRA1A	G	0.097	0.041	2.532	7.37x10^-5^	0.125	0.041	3.374	8.69x10^-7^
rs7861665	9	LOC101929330, GLIS3 (3 kb)	T	0.046	0.106	0.406	5.35x10^-5^	0.051	0.106	0.448	0.00339
rs17054974	9	SEMA4D (51 kb) | GADD45G (55 kb)	C	0.071	0.036	2.064	0.00497	0.098	0.036	2.922	7.04x10^-5^
rs12573965	11	KCNQ1	C	0.129	0.084	1.601	0.01062	0.170	0.084	2.217	6.70x10^-5^
rs7947391	11	NPAS4	A	0.338	0.441	0.649	0.00018	0.354	0.441	0.696	0.00910
rs11613185	12	BCL2L14	A	0.125	0.214	0.526	2.61x10^-5^	0.125	0.214	0.525	0.00064
rs12821816	12	NDUFA12	G	0.419	0.523	0.657	0.00019	0.435	0.523	0.700	0.00829
rs61940911	12	ANKRD13A	T	0.098	0.044	2.386	0.00015	0.104	0.044	2.542	0.00026
rs73195844	12	CCDC63 | MYL2 (4 kb)	T	0.116	0.052	2.411	3.43x10^-5^	0.116	0.052	2.415	0.00025
rs9543114	13	DIS3	C	0.075	0.030	2.635	0.00031	0.098	0.030	3.560	5.93x10^-6^
rs9301108	13	DAOA (653 kb) | EFNB2 (346 kb)	C	0.067	0.033	2.105	0.00547	0.095	0.033	3.103	4.33x10^-5^
rs214003	14	NRXN3	G	0.132	0.080	1.752	0.00253	0.158	0.080	2.163	0.00017
rs4776471	15	RPLP1 (245 kb) | TLE3 (348 kb)	T	0.457	0.338	1.653	1.32x10^-5^	0.464	0.338	1.701	0.00011
rs11856981	15	RPLP1 (288 kb) | TLE3 (305 kb)	T	0.544	0.422	1.638	1.24x10^-5^	0.557	0.422	1.720	6.17x10^-5^
rs4288991	16	TOX3 (91 kb) | CHD9 (416 kb)	C	0.194	0.286	0.600	0.00012	0.196	0.286	0.610	0.00231
rs8065364	17	CARD14	C	0.268	0.366	0.636	0.00019	0.268	0.366	0.635	0.00206
rs578211	18	MYO5B	A	0.303	0.205	1.691	5.49x10^-5^	0.310	0.205	1.742	0.00027
rs4808571	19	MYO9B	A	0.229	0.155	1.619	0.00082	0.268	0.155	1.999	2.13x10^-5^
rs62135557	19	MAU2	G	0.092	0.039	2.494	0.00013	0.074	0.039	1.977	0.01734

*The gene located nearest to the SNP. Distance to the gene in kilo bases (1,000 bp) is shown in parentheses. If no distance is shown, the SNP is located within the gene locus.

World-class athletes defined as endurance athletes (N = 168) with VO_2_max ≥78 ml O_2_/kg/min. Total number of controls = 320.

OR = Odds ratio.

The strongest evidence of association (1.2x10^-5^ < p < 6.1x10^-5^) was detected with a cluster of SNPs located on the long arm of chromosome 15 (15q23) about 69.7 million base pairs from the start of the chromosome (Panel A in Fig 1). While the cluster is located in an intergenic region, the same chromosomal region has been previously reported to be associated with atrial fibrillation in the Framingham Heart Study [35]. Five additional SNPs showed associations with p-values ranging from 2.61x10^-5^ to 7.37x10^-5^; these markers were located within or in the vicinity of genes encoding adrenoceptor alpha 1 A (*ADRA1A*), BCL2-like 14 [apoptosis facilitator] (*BCL2L14*), GLIS family zinc finger 3 (GLIS3), myosin, light-chain 2, regulatory, cardiac, slow (*MYL2*), and myosin VB (*MYO5B*).

To evaluate whether these genetic associations were even more prominent among elite athletes with the highest cardiorespiratory fitness level, the analyses were repeated by comparing only those athletes with a VO_2_max of ≥78 mL/kg/min (N = 168) versus controls. The rationale was that sequence variants playing a role in maximal endurance capacity should cluster even more tightly in world-class endurance athletes compared to endurance athletes with lower VO_2_max levels. An overview of the results is shown Panel B of Fig 1. A total of 50 SNPs showed associations with p-values less than 5.0 x 10^−4^ and eight SNPs with p-values less than 9.7 x 10^−5^. The strongest evidence of association was detected with a SNP located in the *ADRA1A* locus (p = 8.69 x 10^−7^) and the top SNPs are also listed in Table 2.

### Japanese Endurance GWAS

**Fig 2:**
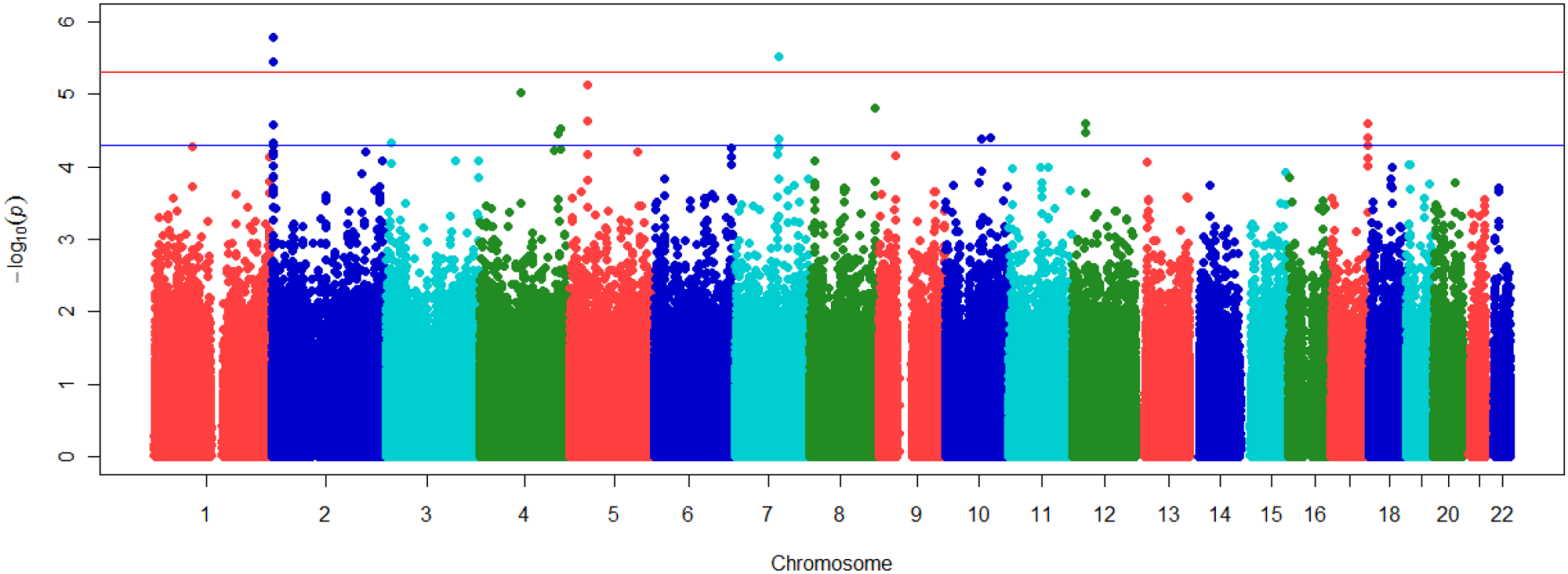
Manhattan plot of -log10(p) values against genomic position for association of elite endurance status with markers in 22 autosomes in Japanese. Red line refers to p = 5x10-6; blue line refers to p = 5x10-5.

In the Japanese cohort, after removing SNPs and individuals failing quality control, 541,179 autosomal SNPs were available for analysis. A total of 31% of the SNPs were common to the Illumina chips used in the Japanese endurance athlete cohort and GENATHLETE. Allelic association analyses were performed by comparing allele-frequency differences between Japanese endurance athletes and controls. The Quantile-Quantile *p*-value plot of observed versus expected—log_10_*(p)* values is shown in S2 Fig. The genomic inflation factor (λ) value in Japanese was 1.002, indicating that there was no substantial evidence of population stratification. A Manhattan plot of—log_10_*(p)* values for associations of elite Japanese endurance status with markers in 22 autosomes is shown in Fig 2.

No SNPs exceeded the threshold for genome-wide significance (*p* value < 5 x 10^−8^). The association results for markers with *p* < 5 x 10^−5^ plus a few markers as defined next are displayed in Table 3. Out of the total SNPs entered into the association analysis, 21 met this threshold. Among them, 3 SNPs were associated at *p* < 5 x 10^−6^. Regional association plots of the top signals were created using LocusZoom Version 1.1 [36], including information on the location and orientation of genes, local estimates of recombination rates and levels of linkage disequilibrium (LD). An individual plot was specified by the SNP of interest and treated as the key marker for the region. Markers within a 500 kb flanking region each side of the index SNP were included. Plots were generated based on Human Genome19 (hg19). Pairwise LD between the index SNP and the surrounding SNPs and recombination rates were estimated in LocusZoom using 1000G Mar 2012 ASN as the reference population.

**Table 3:** Association Results for Markers Chosen for Replication from the Japanese World-Class Endurance Athlete Cohort.

SNP	Chr	Gene*	Minor allele	Freq. in athletes	Freq. in controls	OR	95% CI	p-value
**rs10874242**	**1**	**LPHN2 (153 kb)**	**T**	**0.30**	**0.53**	**0.39**	**0.24–0.62**	**5.38x10**^**-5**^
**s12047209**	**1**	**AKT3**	**C**	**0.42**	**0.22**	**2.60**	**1.61–4.20**	**7.29x10**^**-5**^
**rs921665**	**2**	**TSSC1 (18 kb)**	**G**	**0.30**	**0.57**	**0.32**	**0.20–0.52**	**1.68x10**^**-6**^
**rs2694093**	**2**	**TSSC1**	**G**	**0.17**	**0.36**	**0.35**	**0.20–0.61**	**0.0001**
**rs6548153**	**2**	**TSSC1**	**C**	**0.28**	**0.51**	**0.37**	**0.23–0.59**	**2.69x10**^**-5**^
**rs2361506**	**2**	**MROH2A**	**T**	**0.12**	**0.31**	**0.30**	**0.16–0.56**	**8.32x10**^**-5**^
**rs7650685**	**3**	**VGLL4**	**G**	**0.63**	**0.40**	**2.54**	**1.61–3.99**	**4.73x10**^**-5**^
**rs10007111**	**4**	**MEPE**	**A**	**0.45**	**0.22**	**2.87**	**1.79–4.62**	**9.72x10**^**-6**^
**rs558129**	**4**	**GALNTL6**	**T**	**0.06**	**0.24**	**0.20**	**0.09–0.45**	**3.01x10**^**-5**^
**rs2910756**	**5**	**GDNF-AS1**	**G**	**0.14**	**0.37**	**0.28**	**0.16–0.50**	**7.58x10**^**-6**^
**rs9355947**	**6**	**PARK2**	**G**	**0.57**	**0.34**	**2.52**	**1.60–3.96**	**5.46x10**^**-5**^
**rs6959675**	**7**	**CFAP69**	**C**	**0.27**	**0.49**	**0.38**	**0.24–0.62**	**6.82x10**^**-5**^
**rs11975386**	**7**	**BET1 (70 kb)**	**A**	**0.42**	**0.19**	**3.14**	**1.92–5.13**	**3.07x10**^**-6**^
**rs16906888**	**8**	**FAM135B (898 kb)**	**G**	**0.38**	**0.17**	**2.97**	**1.79–4.92**	**1.58x10**^**-5**^
**rs3780169**	**9**	**PAX5**	**C**	**0.28**	**0.50**	**0.39**	**0.24–0.62**	**7.17x10**^**-5**^
**rs17690338**	**10**	**ZNF503-AS1**	**A**	**0.45**	**0.24**	**2.63**	**1.65–4.21**	**4.21x10**^**-5**^
**rs2761291**	**10**	**MYOF**	**A**	**0.24**	**0.08**	**3.54**	**1.89–6.64**	**4.03x10**^**-5**^
**rs9580890**	**13**	**SPATA13**	**G**	**0.18**	**0.38**	**0.35**	**0.20–0.60**	**8.50x10**^**-5**^
**rs4541108**	**17**	**RBFOX3**	**A**	**0.22**	**0.06**	**4.00**	**2.02–7.90**	**2.51x10**^**-5**^

OR = Odds ratio; CI = 95% confidence interval.

For athletes, N = 60 and for controls, N = 116.

*The gene located nearest to the SNP. Distance to the gene in kilo bases (1,000 bp) is shown in parentheses. If no distance is shown, the SNP is located within the gene locus.

Regional association plots of the 21 SNPs with a *p* < 5 x 10^−5^ were further inspected, leading to the exclusion of 10 SNPs, which were considered as redundant. The regional association plots for the remaining 11 SNPs (see Table 3) are presented in S3–S13 Figs of the Supporting Information. Another set of 7 SNPs with a *p* < 10^−4^ were added to the panel after examination of regional association plots (S14–S20 Figs). Furthermore, rs2694093 (*p* = 0.00014) was independently associated with athlete status and is in a region near the strongest signal rs921665 (chr2:3174321, hg19/1000 Genomes Mar 2012 ASN; S21 Fig). It was also included in the discovery set. All 19 SNPs retained for the replication phase based on the results on the Japanese athletes are summarized in Table 3. These 19 SNPs were combined with the 26 retained from GENATHLETE to constitute the panel of 45 SNPs that was used in the replication cohorts.

### Replication Phase

Table 4 depicts the association results for the 42 SNPs that could be tested in at least two of the four replication cohorts of Caucasians, namely Australian, Polish, Russian and Spanish endurance athletes and controls. Meta-analysis showed that none of the markers summarized in Table 4 reached statistical significance after accounting for multiple testing (Bonferroni-corrected statistical significance p<0.0011), although one marker (rs558129 in *GALNT6*) showed a nominal p-value of P = 0.02. Five SNPs were nominally (p<0.05) associated with endurance athlete status in the Australian study, as well as one in the Polish cohort, four in the Spanish cohort, and five in the Russian cohort. One SNP (rs7947391) showed nominally significant associations in three replication cohorts (Australians, Polish and Spanish). However, as is evident from the meta-analysis results, the direction of the association was not the same across the three studies; the minor allele frequency was lower in athletes than in controls in the Spanish cohort (as well as in the GENATHLETE discovery cohort), whereas Australian and Polish athletes had greater minor allele frequency than their non-athlete controls.

**Table 4:** Results for Replication Meta-Analysis and for Individual Cohorts for All Athletes and Controls of European Descent; Results in bold type are for p≤0.05.

					Meta-analysis				Australia		Poland		Spain		Russia	
SNP	Chr	MAP	Gene*	Effect allele	OR	P	Q	I^2^	OR	P	OR	P	OR	P	OR	P
rs10874242	1	81,426,166	LPHN2 (153 kb)	T	0.95	0.61	0.41	0.0	1.16	0.37	0.86	0.53	0.97	0.88	0.77	0.17
rs17459307	1	162,245,878	NOS1AP	A	1.01	0.95	0.69	0.0	1.08	0.77	1.19	0.61	0.83	0.53	NA	NA
rs12047209	1	243,791,105	AKT3	C	1.19	0.18	0.23	30.5	1.16	0.44	1.07	0.83	0.89	0.63	1.60	**0.009**
rs921665	2	3,170,550	TSSC1 (18 kb)	A	1.29	0.07	0.27	23.0	0.90	0.64	1.56	0.12	1.38	0.20	1.56	**0.05**
rs2694093	2	3,254,403	TSSC1	G	0.87	0.34	0.01	74.1	1.03	0.82	0.93	0.71	1.04	0.81	0.58	**0.00008**
rs6548153	2	3,322,274	TSSC1	A	1.08	0.68	0.06	63.7	0.76	0.13	1.36	0.22	NA	NA	1.28	0.16
rs2118908	2	111,067,015	ACOXL	G	1.05	0.75	0.08	55.7	0.92	0.70	0.68	0.17	1.11	0.64	1.51	**0.02**
rs2361506	2	233,830,694	MROH2A	T	1.13	0.16	0.84	0.0	1.17	0.25	1.02	0.90	NA	NA	1.17	0.33
rs7650685	3	11,660,982	VGLL4	G	0.96	0.57	0.47	0.0	0.84	0.23	0.84	0.38	1.04	0.80	1.13	0.45
rs6799372	3	74,481,495	CNTN3	A	0.90	0.44	0.17	44.0	0.92	0.56	1.17	0.45	0.69	**0.049**	NA	NA
rs10938202	4	42,650,320	ATP8A1	C	1.18	0.38	0.22	31.7	1.83	**0.04**	0.87	0.69	1.37	0.36	0.86	0.61
rs10007111	4	87,828,549	MEPE	A	0.95	0.49	0.96	0.0	0.95	0.70	0.99	0.96	0.89	0.45	0.98	0.90
rs4699824	4	100,801,328	EMCN (283 kb), PPP3CA (222 kb)	G	1.01	0.94	0.07	58.5	0.74	**0.03**	1.02	0.90	1.19	0.28	1.20	0.26
rs558129	4	171,829,960	GALNTL6	T	0.84	**0.02**	0.38	2.1	0.69	**0.01**	0.82	0.31	0.99	0.96	0.89	0.39
rs2910756	5	37,859,972	GDNF-AS1	G	0.92	0.50	0.18	38.2	1.03	0.88	0.95	0.84	0.60	**0.02**	1.08	0.65
rs10499127	6	124,594,509	NKAIN2	G	1.16	0.21	0.60	0.0	1.11	0.62	0.87	0.61	1.38	0.17	1.29	0.30
rs9355947	6	161,887,402	PARK2	A	0.94	0.50	0.24	28.5	0.87	0.27	0.74	0.10	1.15	0.36	1.03	0.86
rs6959675	7	90,253,815	CFAP69	T	0.78	0.49	0.32	11.9	0.36	0.10	0.71	0.69	NA	NA	1.16	0.73
rs11975386	7	94,075,721	BET1 (70 kb)	A	1.24	0.25	0.42	0.0	1.68	0.07	0.71	0.46	1.27	0.55	1.00	0.99
rs17055965	8	26,769,562	ADRA1A	G	1.25	0.13	0.68	0.0	1.43	0.20	0.76	0.53	1.31	0.26	1.26	0.44
rs16906888	8	137,231,375	FAM135B (898 kb)	G	1.17	0.17	0.50	0.0	NA	NA	NA	NA	1.27	0.15	1.09	0.61
rs7861665	9	4,302,988	LOC101929330, GLIS3 (3 kb)	T	0.96	0.77	0.26	25.1	0.82	0.38	0.70	0.28	1.41	0.15	0.92	0.77
rs3780169	9	36,979,398	PAX5	T	1.02	0.76	0.68	0.0	0.90	0.44	1.09	0.63	1.15	0.40	1.04	0.78
rs17690338	10	75,357,798	ZNF503-AS1	A	0.94	0.54	0.23	31.2	1.08	0.56	1.00	0.98	1.00	0.99	0.70	**0.03**
rs2761291	10	93,328,423	MYOF	C	0.90	0.32	0.44	0.0	1.04	0.78	0.76	0.18	NA	NA	0.86	0.37
rs12573965	11	2,660,402	KCNQ1	C	0.76	0.06	0.60	0.0	0.87	0.50	0.75	0.37	0.62	0.06	NA	NA
rs7947391	11	66,419,411	NPAS4	A	1.13	0.57	0.00	87.4	1.37	**0.02**	1.85	**0.0008**	0.63	**0.003**	1.06	0.74
rs11613185	12	12,075,733	BCL2L14	A	0.91	0.62	0.19	41.5	1.07	0.70	0.72	0.18	NA	NA	NA	NA
rs61940911	12	110,029,339	ANKRD13A	T	1.21	0.24	0.95	0.0	1.29	0.31	1.14	0.70	1.17	0.58	NA	NA
rs73195844	12	110,906,768	CCDC63, MYL2 (4 kb)	T	0.86	0.41	0.57	0.0	0.78	0.33	0.96	0.87	NA	NA	NA	NA
rs9580890	13	24,165,281	SPATA13	G	0.87	0.35	0.76	0.0	0.80	0.26	0.89	0.70	NA	NA	1.03	0.92
rs9543114	13	72,767,956	DIS3	C	0.94	0.77	0.61	0.0	0.75	0.38	1.01	0.99	1.29	0.57	NA	NA
rs9301108	13	106,143,743	DAOA (653 kb), EFNB2 (346 kb)	C	1.14	0.45	0.36	6.2	0.75	0.33	1.61	0.21	1.17	0.56	1.44	0.33
rs214003	14	78,336,687	NRXN3	G	0.89	0.55	0.11	50.6	0.59	**0.03**	0.80	0.51	1.52	0.17	0.95	0.83
rs4776471	15	69,700,119	RPLP1 (245 kb), TLE3 (348 kb)	T	1.14	0.25	0.08	55.1	1.17	0.25	1.17	0.39	1.47	**0.01**	0.86	0.29
rs11856981	15	69,743,207	RPLP1 (288 kb), TLE3 (305 kb)	T	1.11	0.15	0.77	0.0	1.08	0.57	1.09	0.65	1.27	0.11	0.96	0.76
rs4288991	16	52,639,252	TOX3 (91 kb), CHD9 (416 kb)	C	0.86	0.09	0.96	0.0	0.89	0.43	0.81	0.38	0.82	0.23	0.91	0.58
rs4541108	17	79,332,527	RBFOX3	A	1.05	0.73	0.99	0.0	1.03	0.90	1.07	0.83	NA	NA	1.06	0.78
rs8065364	17	80,189,160	CARD14	C	0.96	0.62	0.65	0.0	1.00	0.98	1.16	0.45	0.85	0.31	0.92	0.58
rs578211	18	49,868,025	MYO5B	A	1.00	0.98	0.08	59.9	0.83	0.19	0.88	0.56	1.38	0.08	NA	NA
rs4808571	19	17,115,419	MYO9B	A	1.03	0.85	0.06	59.9	0.74	0.08	1.17	0.49	1.49	**0.05**	0.94	0.70
rs62135557	19	19,355,484	MAU2	G	0.93	0.75	0.24	30.7	0.77	0.40	0.61	0.28	1.31	0.30	NA	NA

Effect allele (i.e., minor allele).

OR = odds ratio from a random-effects meta-analysis.

P = p-value for OR from a random-effects meta-analysis.

Q = p-value for Cochrane's Q statistic (tests heterogeneity in effects across individual studies).

I^2^ (I-squared) = heterogeneity index (0–100; 0 = no heterogeneity, 100 = max. heterogeneity).

MAP based on GRCh38 (hg38).

*The gene located nearest to the SNP. Distance to the gene in kilo bases (1,000 bp) is shown in parentheses. If no distance is shown, the SNP is located within the gene locus.

The meta-analysis of the subgroup of athletes classified as world class and truly elite endurance athletes did not reveal any significant associations. Five SNPs had nominal p-values less than 0.05 in Australian world-class athletes, one in Polish world-class athletes, four SNPs in Spanish world-class athletes, and three in Russian world-class athletes (Table 5). However, these associations were generally not directionally consistent among cohorts.

**Table 5:** Results for Meta-Analysis of World-Class Athletes and Controls of European Descent; Results in bold type are for p≤0.05.

					Meta-analysis				Australia		Poland		Spain		Russia	
SNP	Chr	MAP	Gene*	Effect allele	OR	P	Q	I^2^	OR	P	OR	P	OR	P	OR	P
rs10874242	1	81,426,166	LPHN2 (153 kb)	T	1.00	1.00	0.33	11.9	1.19	0.32	1.13	0.68	0.97	0.88	0.67	0.13
rs17459307	1	162,245,878	NOS1AP	A	1.02	0.91	0.60	0.0	1.25	0.43	1.00	0.99	0.83	0.53	NA	NA
rs12047209	1	243,791,105	AKT3	C	1.26	0.31	0.02	69.4	1.16	0.46	0.96	0.92	0.89	0.63	2.29	**0.0002**
rs921665	2	3,170,550	TSSC1 (18 kb)	A	1.19	0.21	0.58	0.0	0.93	0.76	1.13	0.76	1.38	0.20	1.46	0.19
rs2694093	2	3,254,403	TSSC1	G	0.91	0.33	0.32	14.0	0.98	0.91	0.89	0.63	1.04	0.81	0.67	**0.04**
rs6548153	2	3,322,274	TSSC1	A	1.31	0.28	0.03	71.4	0.85	0.40	1.65	0.08	NA	NA	1.71	**0.01**
rs2118908	2	111,067,015	ACOXL	G	1.00	1.00	0.28	20.9	0.98	0.92	0.50	0.08	1.11	0.64	1.23	0.41
rs2361506	2	233,830,694	MROH2A	T	1.12	0.27	0.80	0.0	1.13	0.41	0.99	0.97	NA	NA	1.22	0.32
rs7650685	3	11,660,982	VGLL4	G	0.93	0.40	0.77	0.0	0.82	0.20	0.96	0.86	1.04	0.80	0.91	0.68
rs6799372	3	74,481,495	CNTN3	A	0.86	0.30	0.19	39.3	0.85	0.30	1.23	0.42	0.69	**0.049**	NA	NA
rs10938202	4	42,650,320	ATP8A1	C	1.35	0.08	0.52	0.0	1.90	**0.03**	1.02	0.96	1.37	0.36	1.03	0.94
rs10007111	4	87,828,549	MEPE	A	0.92	0.36	0.99	0.0	0.93	0.61	0.96	0.85	0.89	0.45	0.95	0.82
rs4699824	4	100,801,328	EMCN (283 kb), PPP3CA (222 kb)	G	1.08	0.63	0.04	64.7	0.73	**0.04**	1.24	0.35	1.19	0.28	1.36	0.14
rs558129	4	171,829,960	GALNTL6	T	0.87	0.13	0.78	0.0	0.80	0.15	0.80	0.37	0.99	0.96	0.86	0.47
rs2910756	5	37,859,972	GDNF-AS1	G	0.88	0.36	0.22	32.4	1.10	0.60	0.89	0.73	0.60	**0.02**	0.95	0.83
rs10499127	6	124,594,509	NKAIN2	G	1.25	0.09	0.62	0.0	1.26	0.28	0.83	0.59	1.38	0.17	1.43	0.24
rs9355947	6	161,887,402	PARK2	A	0.95	0.57	0.29	19.1	0.80	0.11	0.83	0.40	1.15	0.36	1.05	0.82
rs6959675	7	90,253,815	CFAP69	T	0.79	0.55	0.54	0.0	0.46	0.23	0.67	0.72	NA	NA	1.18	0.76
rs11975386	7	94,075,721	BET1 (70 kb)	A	1.35	0.14	0.50	0.0	1.82	**0.045**	1.01	0.99	1.27	0.55	0.75	0.61
rs17055965	8	26,769,562	ADRA1A	G	1.20	0.27	0.60	0.0	1.39	0.27	0.91	0.86	1.31	0.26	0.67	0.44
rs16906888	8	137,231,375	FAM135B (898 kb)	G	1.10	0.58	0.20	38.7	NA	NA	NA	NA	1.27	0.15	0.90	0.62
rs7861665	9	4,302,988	LOC101929330, GLIS3 (3 kb)	T	1.00	0.99	0.12	48.3	0.71	0.19	0.62	0.27	1.41	0.15	1.40	0.34
rs3780169	9	36,979,398	PAX5	T	1.07	0.48	0.71	0.0	0.98	0.88	1.27	0.28	1.15	0.40	0.97	0.88
rs17690338	10	75,357,798	ZNF503-AS1	A	0.95	0.62	0.25	27.6	1.13	0.41	0.91	0.69	NA	NA	0.66	0.06
rs2761291	10	93,328,423	MYOF	C	0.84	0.36	0.12	52.8	1.13	0.48	0.72	0.23	NA	NA	0.66	0.07
rs12573965	11	2,660,402	KCNQ1	C	0.79	0.13	0.50	0.0	0.89	0.60	0.95	0.89	0.62	0.06	NA	NA
rs7947391	11	66,419,411	NPAS4	A	1.17	0.51	0.00	85.5	1.35	**0.04**	1.86	**0.007**	0.63	**0.003**	1.23	0.30
rs11613185	12	12,075,733	BCL2L14	A	0.97	0.89	0.24	28.9	1.13	0.53	0.74	0.31	NA	NA	NA	NA
rs61940911	12	110,029,339	ANKRD13A	T	1.12	0.54	0.83	0.0	1.17	0.56	0.87	0.76	1.17	0.58	NA	NA
rs73195844	12	110,906,768	CCDC63, MYL2 (4 kb)	T	0.73	0.16	0.94	0.0	0.74	0.29	0.71	0.35	NA	NA	NA	NA
rs9580890	13	24,165,281	SPATA13	G	0.78	0.16	0.72	0.0	0.78	0.25	0.60	0.24	NA	NA	0.96	0.92
rs9543114	13	72,767,956	DIS3	C	1.03	0.90	0.82	0.0	0.98	0.94	0.86	0.77	1.29	0.57	NA	NA
rs9301108	13	106,143,743	DAOA (653 kb), EFNB2 (346 kb)	C	1.07	0.80	0.08	55.2	0.50	0.06	2.06	0.09	1.17	0.56	1.21	0.71
rs214003	14	78,336,687	NRXN3	G	0.82	0.43	0.07	57.4	0.51	**0.01**	0.82	0.65	1.52	0.17	0.73	0.34
rs4776471	15	69,700,119	RPLP1 (245 kb), TLE3 (348 kb)	T	1.09	0.59	0.02	68.3	1.23	0.15	1.03	0.89	1.47	**0.01**	0.70	0.07
rs11856981	15	69,743,207	RPLP1 (288 kb), TLE3 (305 kb)	T	1.09	0.30	0.53	0.0	1.10	0.50	0.88	0.59	1.27	0.11	0.98	0.91
rs4288991	16	52,639,252	TOX3 (91 kb), CHD9 (416 kb)	C	0.85	0.09	0.85	0.0	0.85	0.33	0.70	0.25	0.82	0.23	0.98	0.92
rs4541108	17	79,332,527	RBFOX3	A	1.16	0.34	0.87	0.0	1.06	0.77	1.22	0.56	NA	NA	1.27	0.37
rs8065364	17	80,189,160	CARD14	C	0.95	0.59	0.83	0.0	1.03	0.84	0.93	0.77	0.85	0.31	1.02	0.94
rs578211	18	49,868,025	MYO5B	A	0.94	0.77	0.04	69.2	0.77	0.10	0.74	0.30	1.38	0.08	NA	NA
rs4808571	19	17,115,419	MYO9B	A	1.05	0.73	0.16	42.6	0.80	0.22	0.97	0.92	1.49	**0.05**	1.06	0.78
rs62135557	19	19,355,484	MAU2	G	0.89	0.71	0.15	47.1	0.79	0.46	0.33	0.12	1.31	0.30	NA	NA

Effect allele (i.e., minor allele).

OR = odds ratio from a random-effects meta-analysis.

P = p-value for OR from a random-effects meta-analysis.

Q = p-value for Cochrane's Q statistic (tests heterogeneity in effects across individual studies).

I^2^ (I-squared) = heterogeneity index (0–100; 0 = no heterogeneity, 100 = max. heterogeneity).

MAP based on GRCh38 (hg38).

*The gene located nearest to the SNP. Distance to the gene in kilo bases (1,000 bp) is shown in parentheses. If no distance is shown, the SNP is located within the gene locus.

Next, we report on the associations between 35 SNPs identified in the discovery phase and endurance athlete status in Ethiopian and Kenyan athletes and controls (Table 6). The lower number of SNPs available for these analyses is due to the fact that several SNPs selected for the replication phase were monomorphic or very rare in African populations. The meta-analysis of the Ethiopian and Kenyan athletes did not reveal any associations that were significant after accounting for multiple testing, although four SNPs had nominal p-value less than 0.05. Five and three SNPs had nominal p-value less than 0.05 in the Kenyan sample (276 athletes versus 83 controls) and in the Ethiopian study (75 athletes versus 198 controls), respectively, but none of these SNPs overlapped between the two cohorts. In fact, the most significant SNPs observed in the Kenyan cohort showed associations in the opposite direction in the Ethiopian cohort.

**Table 6:** Results for the Replication in Athletes and Controls from Kenya and Ethiopia; Results in bold type are for p ≤0.05.

					Meta-analysis				Kenya		Ethiopia	
SNP	Chr	MAP	Gene*	Effect allele	OR	P	Q	I^2^	OR	P	OR	P
rs10874242	1	81,426,166	LPHN2 (153 kb)	T	1.39	0.11	0.22	32.9	1.14	0.58	1.70	**0.02**
rs12047209	1	243,791,105	AKT3	C	0.74	0.67	0.02	82.5	0.37	**0.01**	1.52	0.33
rs921665	2	3,170,550	TSSC1 (18 kb)	A	0.75	**0.03**	0.33	0.0	0.67	**0.02**	0.87	0.51
rs2694093	2	3,254,403	TSSC1	G	0.94	0.70	0.84	0.0	0.97	0.89	0.91	0.68
rs6726044	2	68,911,860	BMP10 (40 kb)	A	1.07	0.85	0.02	81.7	1.57	**0.04**	0.73	0.19
rs2118908	2	111,067,015	ACOXL	G	0.98	0.94	0.98	0.0	0.98	0.94	0.99	0.98
rs7650685	3	11,660,982	VGLL4	G	0.98	0.90	0.61	0.0	1.05	0.80	0.92	0.65
rs6799372	3	74,481,495	CNTN3	A	1.39	**0.04**	0.92	0.0	1.37	0.16	1.42	0.13
rs10938202	4	42,650,320	ATP8A1	C	0.57	0.07	0.21	35.4	0.74	0.31	0.40	**0.02**
rs10007111	4	87,828,549	MEPE	A	1.03	0.95	0.02	82.2	1.51	**0.05**	0.68	0.14
rs4699824	4	100,801,328	EMCN (283 kb), PPP3CA (222 kb)	A	1.00	0.98	0.64	0.0	1.07	0.74	0.94	0.73
rs558129	4	171,829,960	GALNTL6	T	0.80	0.15	0.44	0.0	0.72	0.11	0.91	0.69
rs2910756	5	37,859,972	GDNF-AS1	G	0.74	**0.04**	0.35	0.0	0.82	0.27	0.62	**0.04**
rs10499127	6	124,594,509	NKAIN2	G	0.92	0.67	0.37	0.0	0.75	0.34	1.08	0.78
rs9355947	6	161,887,402	PARK2	G	0.93	0.60	0.73	0.0	0.89	0.54	0.98	0.90
rs11975386	7	94,075,721	BET1 (70 kb)	A	1.05	0.83	0.83	0.0	1.09	0.76	0.99	0.98
rs17055965	8	26,769,562	ADRA1A	G	1.27	0.37	0.70	0.0	1.44	0.39	1.17	0.64
rs16906888	8	137,231,375	FAM135B (898 kb)	G	1.12	0.40	0.46	0.0	1.23	0.27	1.01	0.98
rs7861665	9	4,302,988	LOC101929330, GLIS3 (3 kb)	T	0.81	0.13	0.70	0.0	0.78	0.16	0.87	0.51
rs3780169	9	36,979,398	PAX5	T	0.80	0.12	0.32	0.0	0.90	0.59	0.68	0.08
rs17054974	9	89,549,371	SEMA4D (51 kb), GADD45G (55 kb)	C	1.08	0.87	0.13	57.4	1.75	0.21	0.71	0.36
rs17690338	10	75,357,798	ZNF503-AS1	A	1.02	0.92	0.19	42.4	1.24	0.31	0.84	0.40
rs12573965	11	2,660,402	KCNQ1	C	0.82	0.90	0.01	86.3	0.19	**0.005**	4.02	0.10
rs7947391	11	66,419,411	NPAS4	G	0.75	**0.03**	0.71	0.0	0.71	0.06	0.79	0.21
rs61940911	12	110,029,339	ANKRD13A	T	1.23	0.57	0.69	0.0	0.98	0.98	1.34	0.48
rs73195844	12	110,906,768	CCDC63, MYL2 (4 kb)	T	1.43	0.29	0.93	0.0	1.51	0.59	1.41	0.35
rs9543114	13	72,767,956	DIS3	C	1.37	0.56	0.92	0.0	1.51	0.71	1.33	0.65
rs9301108	13	106,143,743	DAOA (653 kb), EFNB2 (346 kb)	C	0.82	0.69	0.00	87.9	0.50	**0.0008**	1.38	0.26
rs214003	14	78,336,687	NRXN3	G	0.86	0.38	0.21	36.3	0.74	0.09	1.04	0.86
rs4776471	15	69,700,119	RPLP1 (245 kb), TLE3 (348 kb)	T	1.12	0.38	0.85	0.0	1.10	0.62	1.15	0.46
rs11856981	15	69,743,207	RPLP1 (288 kb), TLE3 (305 kb)	T	1.13	0.37	0.98	0.0	1.13	0.54	1.14	0.51
rs4288991	16	52,639,252	TOX3 (91 kb), CHD9 (416 kb)	C	0.96	0.78	0.72	0.0	1.03	0.92	0.91	0.66
rs8065364	17	80,189,160	CARD14	C	1.24	0.13	0.74	0.0	1.30	0.20	1.19	0.39
rs578211	18	49,868,025	MYO5B	A	0.80	0.15	0.40	0.0	0.88	0.51	0.67	0.12
rs4808571	19	17,115,419	MYO9B	A	0.94	0.89	0.53	0.0	1.30	0.68	0.77	0.61

OR = odds ratio from a random-effects meta-analysis.

P = p-value for OR from a random-effects meta-analysis.

Q = p-value for Cochrane's Q statistic (tests heterogeneity in effects across individual studies).

I^2^ (I-squared) = heterogeneity index (0–100; 0 = no heterogeneity, 100 = max. heterogeneity).

MAP based on GRCh38 (hg38).

*The gene located nearest to the SNP. Distance to the gene in kilo bases (1,000 bp) is shown in parentheses. If no distance is shown, the SNP is located within the gene locus.

In an attempt to identify lead SNPs based on all the cohorts contributing to the GAMES project, meta-analyses were performed using all contributing studies. The results are summarized in Table 7. Finally, the summary meta-analyses were repeated with all subsamples of world-class endurance athletes and these results are depicted in Table 8. Among all available athletes and controls (9 studies, including discovery cohorts), the meta-analysis revealed one statistically significant SNP (rs558129 at *GALNTL6* locus, p = 0.0002), even after correcting for multiple testing (Bonferroni-corrected statistical significance p<0.0011). As shown by the low heterogeneity index (I^2^ = 0), all eight cohorts showed the same direction of association with rs558129, even though p-values varied considerably across the individual studies. When the meta-analysis was repeated without the discovery cohorts, the association persisted near the multiple testing-adjusted significance threshold (p = 0.0019).

**Table 7:** Results for the Meta-analysis of All Studies of All Athletes and Controls (GENATHLETE, Japan, Australia, Poland, Russia, Spain, Kenya, and Ethiopia); Results in bold type are for p ≤ 0.05.

SNP	Chr	MAP	Gene*	Effect Allele	N	OR	P	Q	I^2^
rs10874242	1	81,426,166	LPHN2 (153 kb)	T	8	1.01	0.94	0.02	57.9
rs17459307	1	162,245,878	NOS1AP	A	6	0.80	0.29	0.03	60.8
rs12047209	1	243,791,105	AKT3	C	8	1.09	0.49	0.01	60.5
rs921665	2	3,170,550	TSSC1 (18 kb)	A	8	1.07	0.57	0.00	67.2
rs2694093	2	3,254,403	TSSC1	G	8	0.85	**0.04**	0.07	47.4
rs6548153	2	3,322,274	TSSC1	A	3	1.08	0.68	0.06	63.7
rs6726044	2	68,911,860	BMP10 (40 kb)	A	5	1.17	0.41	0.00	84.5
rs2118908	2	111,067,015	ACOXL	G	8	1.16	0.20	0.14	36.3
rs2361506	2	233,830,694	MROH2A	T	5	0.97	0.76	0.09	50.3
rs7650685	3	11,660,982	VGLL4	G	8	1.01	0.78	0.66	0.0
rs6799372	3	74,481,495	CNTN3	A	7	0.95	0.66	0.00	70.1
rs10938202	4	42,650,320	ATP8A1	C	8	1.06	0.78	0.00	70.4
rs10007111	4	87,828,549	MEPE	A	8	1.02	0.76	0.13	37.3
rs4699824	4	100,801,328	EMCN (283 kb), PPP3CA (222 kb)	G	8	1.08	0.35	0.02	59.0
rs558129	4	171,829,960	GALNTL6	T	8	0.81	**0.0002**	0.66	0.0
rs2910756	5	37,859,972	GDNF-AS1	G	8	0.89	0.23	0.02	58.8
rs10499127	6	124,594,509	NKAIN2	G	8	1.10	0.48	0.01	63.4
rs9355947	6	161,887,402	PARK2	A	8	0.94	0.22	0.58	0.0
rs6959675	7	90,253,815	CFAP69	T	3	0.78	0.49	0.32	11.9
rs11975386	7	94,075,721	BET1 (70 kb)	A	8	1.15	0.12	0.61	0.0
rs17055965	8	26,769,562	ADRA1A	G	7	1.43	**0.008**	0.21	28.0
rs16906888	8	137,231,375	FAM135B (898 kb)	G	6	1.06	0.39	0.46	0.0
rs7861665	9	4,302,988	LOC101929330, GLIS3 (3 kb)	T	8	0.84	0.22	0.01	62.4
rs3780169	9	36,979,398	PAX5	T	8	1.02	0.81	0.22	26.4
rs17054974	9	89,549,371	SEMA4D (51 kb), GADD45G (55 kb)	C	5	1.32	0.13	0.11	47.4
rs17690338	10	75,357,798	ZNF503-AS1	A	8	0.94	0.24	0.36	9.3
rs2761291	10	93,328,423	MYOF	C	3	0.90	0.32	0.44	0.0
rs12573965	11	2,660,402	KCNQ1	C	7	0.87	0.52	0.00	69.9
rs7947391	11	66,419,411	NPAS4	A	8	1.11	0.46	0.00	85.2
rs11613185	12	12,075,733	BCL2L14	A	2	0.91	0.62	0.19	41.5
rs12821816	12	94,976,701	NDUFA12	G	3	0.88	0.44	0.00	82.0
rs61940911	12	110,029,339	ANKRD13A	T	6	1.45	**0.01**	0.29	19.5
rs73195844	12	110,906,768	CCDC63, MYL2 (4 kb)	T	5	1.29	0.33	0.01	70.5
rs9580890	13	24,165,281	SPATA13	G	3	0.87	0.35	0.76	0.0
rs9543114	13	72,767,956	DIS3	C	6	1.32	0.26	0.09	48.1
rs9301108	13	106,143,743	DAOA (653 kb), EFNB2 (346 kb)	C	7	1.14	0.55	0.00	74.3
rs214003	14	78,336,687	NRXN3	G	8	0.98	0.89	0.01	63.7
rs4776471	15	69,700,119	RPLP1 (245 kb), TLE3 (348 kb)	T	8	1.18	**0.04**	0.01	59.9
rs11856981	15	69,743,207	RPLP1 (288 kb), TLE3 (305 kb)	T	8	1.17	**0.02**	0.09	42.9
rs4288991	16	52,639,252	TOX3 (91 kb) | CHD9 (416 kb)	C	8	0.84	**0.007**	0.31	14.9
rs4541108	17	79,332,527	RBFOX3	A	3	1.05	0.73	0.99	0.0
rs8065364	17	80,189,160	CARD14	C	8	0.95	0.53	0.03	53.8
rs578211	18	49,868,025	MYO5B	A	7	1.00	1.00	0.00	74.2
rs4808571	19	17,115,419	MYO9B	A	8	1.14	0.30	0.03	56.1
rs62135557	19	19,355,484	MAU2	G	4	1.18	0.59	0.01	76.3

N = Number of studies used in meta-analysis.

OR = odds ratio from a random-effects meta-analysis.

P = p-value for OR from a random-effects meta-analysis.

Q = p-value for Cochrane's Q statistic (tests heterogeneity in effects across individual studies).

I^2^ (I-squared) = heterogeneity index (0–100; 0 = no heterogeneity, 100 = max. heterogeneity).

MAP based on GRCh38 (hg38).

*The gene located nearest to the SNP. Distance to the gene in kilo bases (1,000 bp) is shown in parentheses. If no distance is shown, the SNP is located within the gene locus.

**Table 8:** Results for the Meta-analysis of All Studies of World-class Athletes and Controls (GENATHLETE, Japan, Australia, Poland, Russia, Spain); Results in bold type are for p ≤ 0.05.

SNP	Chr	MAP	Gene*	Effect Allele	N	OR	P	Q	I^2^
rs10874242	1	81,426,166	LPHN2 (153 kb)	T	6	0.91	0.50	0.03	59.1
rs17459307	1	162,245,878	NOS1AP	A	5	0.83	0.30	0.25	25.7
rs12047209	1	243,791,105	AKT3	C	6	1.29	0.17	0.00	75.8
rs921665	2	3,170,550	TSSC1 (18 kb)	A	6	1.17	0.31	0.05	55.1
rs2694093	2	3,254,403	TSSC1	G	6	0.86	**0.04**	0.36	9.1
rs6548153	2	3,322,274	TSSC1	A	3	1.31	0.28	0.03	71.4
rs6726044	2	68,911,860	BMP10 (40 kb)	A	3	1.25	0.35	0.00	82.2
rs2118908	2	111,067,015	ACOXL	G	5	1.15	0.48	0.02	63.6
rs2361506	2	233,830,694	MROH2A	T	5	0.88	0.41	0.01	72.9
rs7650685	3	11,660,982	VGLL4	G	6	1.06	0.56	0.06	53.1
rs6799372	3	74,481,495	CNTN3	A	5	0.79	**0.02**	0.19	35.4
rs10938202	4	42,650,320	ATP8A1	C	5	1.56	**0.005**	0.30	17.9
rs10007111	4	87,828,549	MEPE	A	6	1.05	0.68	0.03	58.6
rs4699824	4	100,801,328	EMCN (283 kb), PPP3CA (222 kb)	G	6	1.10	0.53	0.00	77.6
rs558129	4	171,829,960	GALNTL6	T	6	0.78	**0.04**	0.05	54.5
rs2910756	5	37,859,972	GDNF-AS1	G	6	0.88	0.49	0.00	74.5
rs10499127	6	124,594,509	NKAIN2	G	5	1.24	0.25	0.01	67.1
rs9355947	6	161,887,402	PARK2	A	6	0.84	0.11	0.02	61.7
rs6959675	7	90,253,815	CFAP69	T	3	0.79	0.55	0.54	0.0
rs11975386	7	94,075,721	BET1 (70 kb)	A	6	1.23	0.40	0.02	64.1
rs17055965	8	26,769,562	ADRA1A	G	5	1.44	0.18	0.01	69.4
rs16906888	8	137,231,375	FAM135B (898 kb)	G	4	1.15	0.52	0.00	84.5
rs7861665	9	4,302,988	LOC101929330, GLIS3 (3 kb)	T	6	0.94	0.78	0.01	69.8
rs3780169	9	36,979,398	PAX5	T	6	1.12	0.19	0.22	28.1
rs17054974	9	89,549,371	SEMA4D (51 kb), GADD45G (55 kb)	C	3	1.70	0.08	0.04	68.3
rs17690338	10	75,357,798	ZNF503-AS1	A	6	1.03	0.84	0.01	67.5
rs2761291	10	93,328,423	MYOF	C	3	0.84	0.36	0.12	52.8
rs12573965	11	2,660,402	KCNQ1	C	5	1.05	0.84	0.00	77.4
rs7947391	11	66,419,411	NPAS4	A	6	1.16	0.45	0.00	86.5
rs11613185	12	12,075,733	BCL2L14	A	2	0.97	0.89	0.24	28.9
rs12821816	12	94,976,701	NDUFA12	G	2	0.95	0.76	0.03	71.4
rs61940911	12	110,029,339	ANKRD13A	T	4	1.39	0.16	0.07	57.1
rs73195844	12	110,906,768	CCDC63, MYL2 (4 kb)	T	3	1.11	0.81	0.00	84.6
rs9580890	13	24,165,281	SPATA13	G	3	0.78	0.16	0.72	0.0
rs9543114	13	72,767,956	DIS3	C	4	1.47	0.30	0.01	73.1
rs9301108	13	106,143,743	DAOA (653 kb), EFNB2 (346 kb)	C	4	1.37	0.33	0.00	75.8
rs214003	14	78,336,687	NRXN3	G	5	1.07	0.77	0.00	76.4
rs4776471	15	69,700,119	RPLP1 (245 kb), TLE3 (348 kb)	T	5	1.24	0.08	0.01	69.5
rs11856981	15	69,743,207	RPLP1 (288 kb), TLE3 (305 kb)	T	5	1.23	**0.04**	0.05	54.6
rs4288991	16	52,639,252	TOX3 (91 kb), CHD9 (416 kb)	C	6	0.79	**0.003**	0.57	0.0
rs4541108	17	79,332,527	RBFOX3	A	3	1.16	0.34	0.87	0.0
rs8065364	17	80,189,160	CARD14	C	6	0.87	0.10	0.26	22.6
rs578211	18	49,868,025	MYO5B	A	5	1.12	0.53	0.00	76.4
rs4808571	19	17,115,419	MYO9B	A	5	1.24	0.22	0.01	69.2
rs62135557	19	19,355,484	MAU2	G	4	1.11	0.71	0.05	60.7

N = Number of studies used in meta-analysis.

OR = odds ratio from a random-effects meta-analysis.

P = p-value for OR from a random-effects meta-analysis.

Q = p-value for Cochrane's Q statistic (tests heterogeneity in effects across individual studies).

I^2^ (I-squared) = heterogeneity index (0–100; 0 = no heterogeneity, 100 = max. heterogeneity).

MAP based on GRCh38 (hg38).

*The gene located nearest to the SNP. Distance to the gene in kilo bases (1,000 bp) is shown in parentheses. If no distance is shown, the SNP is located within the gene locus.

When the meta-analysis was restricted to the world class athletes versus all controls, none of the SNPs retained for replication reached multiple testing-corrected statistical significance, although SNPs rs4288991 (p = 0.0028) and rs10938202 (p = 0.0053) were trending close. SNP rs558129 was characterized by a nominal p value of 0.04 in this meta-analysis.

## Discussion

It is commonly recognized that to perform at a world-class level in endurance athletic events, one has to be intrinsically well endowed in terms of cardiorespiratory and skeletal muscle potential to exercise at high intensity for sustained periods of time in combination with the ability to respond very favorably to exercise training regimens. Observational genetic epidemiology studies and experimental studies in humans as well as in rodents have shown that there are substantial genetic components to intrinsic cardiorespiratory fitness and its trainability [5, 9, 10]. However, the exact genomic features responsible for these genetic effects have not been identified despite many years of candidate gene driven research, a topic that has been reviewed in recent times [18, 37–39]. A limited number of studies have used an unbiased genomic search to identify genomic regions harboring allelic markers of athlete status [40] or response to exercise training of physiological traits [30, 31, 41–43] or GWAS-identified loci associated with determinants of aerobic fitness or its trainability [28–30] but most findings have not been subjected to replication studies. The present report constitutes the largest effort to date designed to identify in an unbiased manner common variants that could begin to define the world-class endurance performance genotype. Even though the total sample size was substantially higher than any other published effort in this field, it is recognized that it was not optimal for the identification of common genomic variants with small effect size that could discriminate between elite endurance athletes and sedentary controls. The main conclusion from the present study is that common genetic variants do not appear to be strong determinants of elite endurance athlete status.

From the GWAS performed on a panel of Japanese endurance athletes and controls plus the CardioMetabochip screen on the endurance athletes from four countries and their matched controls of the GENATHLETE cohort, a panel of 45 SNPs was retained and carried forward for replication in seven other cohorts. Importantly, none of these 45 SNPs reached genome-wide significance in the two discovery studies. But these studies were small and were expected to generate targets at the genome-wide level of significance only if SNPs with large effect sizes were contributing to elite endurance athlete status. There was no overlap among the top SNPs identified in both discovery panels.

When the results from all cohorts were pooled together in meta-analyses, SNP rs558129 located in the *GALNTL6* locus on chromosome 4q34.1 was statistically significant (p = 0.0002) even after multiple testing was taken into account. The nominal p-values of the individual cohorts ranged from 0.011 (Australia) to 0.9572 (Spain), but the direction of the association was uniform across all cohorts, that is the T allele was less frequent in athletes than in controls. The association remained robust after excluding the discovery cohort from the meta-analysis (p = 0.0071). The same SNP in *GALNTL6* was also nominally significant (p = 0.037) when the meta-analysis was undertaken on the subsets of world-class endurance athletes. This illustrates how random effects meta-analysis helps to identify consistent trends across several relatively small cohorts which individually would not allow for the documentation of such an association. The *GALNTL6* gene encodes N-acetylgalactosaminyltransferase-like 6 but the functional role of the peptide is not fully elucidated at this time. It is expressed primarily in the testes but also in the brain and to some extent in skeletal muscle [44]. The rs558129 polymorphic site is in the last intron of the gene.

A few weak leads may also be of interest in future research. Two SNPs in or near *TSSC1*, the gene encoding tumor suppressing substransferable candidate 1, were associated with endurance athlete status in the cohort from Russia and one of these SNPs (rs2694093) was also shown to be nominally significant and directionally consistent in the global meta-analysis of all cohorts for all endurance athletes as well as the world-class athletes. Of potential interest could also be SNPs near *TOX3/CHD9* on chr 16 and *RPLP1*/*TLE3* on chr 15 which were nominally associated with endurance athlete status in the global meta-analyses of all endurance athletes and in the subset of world-class athletes as well. None of these genes have been implicated in exercise biology before.

Interestingly, SNPs in three candidate genes *(CKM*, *ACTN3 and GNB3*) selected on the basis of prior results in Japanese endurance athletes were not associated with endurance athlete status in any of the cohorts or in meta-analyses. Along the same line, 161 SNPs related to 13 candidate genes for endurance performance derived from the human gene map for physical performance [18] were available among the CardioMetabochip markers genotyped in GENATHLETE. These candidate genes were: *ACE*, *ACSL1*, *ACTN3*, *ADRB1*, *ADRB2*, *AMPD1*, *BDKRB2*, *GH1*, *IL6*, *KDR*, *NOS3*, *PPARA and PPARGC1A*. None of the 161 SNPs reached the Bonferroni-adjusted significance threshold of p = 3.5 x 10^−7^. Two SNPs in the vicinity of *PPARGC1A* were the most strongly associated with endurance athlete status at p≤0.0012. None of the SNP associations were close to significance when the subsample of the GENATHLETE world-class endurance athletes (VO_2_max ≥78 mL O^2^/kg/min) was considered.

Over all, there is no convincing evidence for the contribution of common genomic variants to elite endurance performance in the present report. However, since we were underpowered to identify contributing alleles with small effect sizes, one may be justified in proposing that a few leads, even though supported by very modest levels of evidence, be explored in subsequent studies with adequate statistical power. For instance, the joint contribution from gene-members of a pathway or a network module could be significant, even if individual gene contributions are small and non-significant. We have used this approach successfully in other GWA studies, for example on the analysis of genetic associations to maximal oxygen uptake in response to exercise [45]. An alternative approach employs unsupervised bioinformatics data analysis techniques to generate functional hypotheses on how some of the top candidates from a study may be related to a trait. Graph based methods, such as those based on stochastic random walks [46], are well suited for this type of analysis and can provide useful information for candidate gene prioritization. In this regard, it may be useful to expand the comparison of world-class endurance athletes and sedentary controls to DNA sequence variants in or in the vicinity of a number of genes (for instance: *GALNTL6* but also perhaps *ADRA1A*, *ATP8A1*, *CHD9*, *CNTN3*, *RPLP1*, *TLE3*, *TOX3*, *TSSC1*, *AKT3*, *BET1*, *BMP10*) in order to shed some light on the genetics, biology and the highly demanding selection process leading to world class endurance performance. Thus, using the 12 genes listed above in a Biograph analysis, in which the query was on athletic performance, we have found additional support for the suggestion that sequence variants in these genes should be further investigated (results not shown). Even when SNPs and genes are characterized by small effect size and marginal nominal significance, the joint contributions from gene-members of a pathway or a network may provide useful information.

The present report has several limitations. The SNP chips used for the discovery phase differed between GENATHLETE and the Japanese cohort of endurance athlete. These discovery studies were performed independently and later used to identify the panel of the most promising 45 SNPs. It is also recognized that the CardioMetabochip used in GENATHLETE offers less than a comprehensive coverage of the genome and it may provide less than optimal inclusion of markers and genes that could be important for the peripheral determinants of endurance performance. In some of the replication studies, participants in the control group were from the general population or recreationally active subjects while in others they were confirmed sedentary participants. However, even though the endurance athletes were exposed to variable training regimens, which are undoubtedly heterogeneous by athletic event and country, these factors should not have an influence on the results as they all achieved national and international levels of competition requiring an extraordinary level of cardiorespiratory endurance. Obviously sample size continues to be a major issue for this type of studies. This is a challenge that is very difficult to overcome as the number of world-class endurance athletes on the planet is limited and those who reach this level of performance are hesitant to give consent to participate in genetic studies. Finally, one has also to consider that a number of the athletes who gave consent to participate in these genetic studies are using performance enhancement drugs. However, it is unlikely that doping of any kind could have influenced the results as the use of these drugs is not by itself responsible for an athlete reaching world-class level but rather their uses come more into play when athletes of world-class caliber are engaged competitively against one another.

In all likelihood, attaining the required sample size of world class caliber endurance athletes, for an adequate statistical power in the search for critical sequence variants, will require a buy-in from the relevant world sports federations and the International Olympic Committee. With their support and active participation, thousands of world-class endurance athletes could be enrolled in genomics studies aimed at understanding the fundamentals of inherited biological traits that are necessary to perform at the world class level. Such an effort, particularly if it relied on whole genome sequencing, would allow for the exploration of not only common polymorphisms but also rare variants and copy number variants and could be complemented by the investigation of epigenomic signatures in accessible tissues. In summary, we found that the T allele in *GALNTL6* was less frequent in endurance athletes of all studies compared to ethnicity-matched controls. However, we could not find evidence for a detailed genomic signature that differentiates endurance athletes from controls.

## Supporting Information

S1 Fig Distribution of GENATHLETE VO_2_max of the 315 Elite Endurance Athletes. (TIFF)

S2 Fig Quantile-Quantile plot of observed vs expected—log 10 p values for genome-wide data from Japanese athletes and controls. (TIFF)

S3 Fig Regional association plot of the index SNP—rs921665. (TIF)

S4 Fig Regional association plot of the index SNP—rs6548153. (TIF)

S5 Fig Regional association plot of the index SNP—rs7650685. (TIF)

S6 Fig Regional association plot of the index SNP—rs10007111. (TIF)

S7 Fig Regional association plot of the index SNP—rs558129. (TIF)

S8 Fig Regional association plot of the index SNP—rs2910756. (TIF)

S9 Fig Regional association plot of the index SNP—rs11975386. (TIF)

S10 Fig Regional association plot of the index SNP—rs16906888. (TIF)

S11 Fig Regional association plot of the index SNP—rs17690338. (TIF)

S12 Fig Regional association plot of the index SNP—rs2761291. (TIF)

S13 Fig Regional association plot of the index SNP—rs4541108. (TIF)

S14 Fig Regional association plot of the index SNP—rs10874242. (TIFF)

S15 Fig Regional association plot of the index SNP—rs12047209. (TIFF)

S16 Fig Regional association plot of the index SNP—rs2361506. (TIFF)

S17 Fig Regional association plot of the index SNP—rs9355947. (TIFF)

S18 Fig Regional association plot of the index SNP—rs6959675. (TIFF)

S19 Fig Regional association plot of the index SNP—rs3780169. (TIFF)

S20 Fig Regional association plot of the index SNP—rs9580890. (TIFF)

S21 Fig Regional association plot of the index SNP—rs2694093. (TIFF)

S1 File Supporting Information Text. (DOCX)

S1 Table The Discovery Phase: The GENATHLETE Cohort. (TIFF)
