# Supplementary material for: No Evidence of a Common DNA Variant Profile Specific to World Class Endurance Athletes
Source: PLoS One. 2016 Jan 29;11(1):e0147330. doi: 10.1371/journal.pone.0147330 (PMC4732768; doi:10.1371/journal.pone.0147330)
Supplement: S1 File — (DOCX) [file pone.0147330.s022.docx]

**Supporting Information**

**No Evidence of a Common DNA Variant Profile Specific to World Class Endurance Athletes**

Tuomo Rankinen^1*^, Noriyuki Fuku^2*^, Bernd Wolfarth^3^, Guan Wang^4^, Mark A. Sarzynski^1, 23^, Dmitry G. Alexeev^5^, Ildus I. Ahmetov^5,6^,Marcel R. Boulay^7^, Pawel Cieszczyk^8,9^, Nir Eynon^10^, Maxim L. Filipenko ^11, 12^, Fleur C. Garton^13,14^, Edward V. Generozov^5^, Vadim M. Govorun^5^, Peter J. Houweling^11,12^, Takashi Kawahara^15^, Elena S. Kostryukova^5^, Nickolay A. Kulemin^5^, Andrey K. Larin^5^, Agnieszka Maciejewska-Karłowska^8^, Motohiko Miyachi^16^, Carlos A. Muniesa^17^, Haruka Murakami^16^, Elena A. Ospanova^5^, Sandosh Padmanabhan^18^, Alexander V. Pavlenko^5^, Olga N. Pyankova ^11,12^, Catalina Santiago^17^, Marek Sawczuk^8^, Robert A. Scott^19^, Vladimir V. Uyba^20^, Thomas Yvert^17^, Louis Perusse^7^, Sujoy Ghosh^21^, Rainer Rauramaa^22^, Kathryn N. North^13,14^, Alejandro Lucia^17^, Yannis Pitsiladis^4^, Claude Bouchard^1^

^1^Human Genomics Laboratory, Pennington Biomedical Research Center, Louisiana State University System, Baton Rouge, Louisiana, USA

^2^Graduate School of Health and Sports Science, Juntendo University, Chiba, Japan

^3^Department of Sport Medicine Humboldt University and Charite University School of Medicine, Berlin, Germany

^4^Centre for Sport and Exercise Science and Medicine (SESAME), University of Brighton, Eastbourne, UK

^5^Research Institute for Physical-Chemical Medicine, Moscow, Russia

^6^Sport Technology Research Centre, Volga Region State Academy of Physical Culture, Sport and Tourism, Kazan, Russia

^7^Department of Kinesiology, Laval University, Ste-Foy, Québec, Canada

^8^University of Szczecin, Department of Physical Education and Health Promotion, Szczecin, Poland

^9^Academy of Physical Education and Sport, Department of Tourism and Recreation, Gdansk, Poland

^10^Institute of Sport, Exercise and Active Living (ISEAL), Victoria University, Victoria, Australia

^11^Pharmacogenomics Laboratory, Institute of Chemical Biology and Fundamental Medicine of SB RAS, Novosibirsk, Russia

^12^Novosibirsk State University, Novosibirsk, Russia

^13^Murdoch Childrens Research Institute and Department of Paediatrics, University of Melbourne, Victoria, Australia

^14^Institute of Neuroscience and Muscle Research, Childrens Hospital Westmead, Westmead, Australia

^15^Department of Sports Medicine, Japan Institute of Sports Sciences, Tokyo, Japan

^16^Department of Health Promotion and Exercise, National Institute of Health and Nutrition, Tokyo, Japan

^17^Universidad Europea and Research Institute i+12, Madrid, Spain

^18^College of Medicine, Veterinary & Life Sciences, Institute of Cardiovascular & Medical Sciences, University of Glasgow, Glasgow, UK

^19^MRC Epidemiology Unit, University of Cambridge School of Clinical Medicine, Institute of Metabolic Science, Cambridge Biomedical Campus, Cambridge, UK

^20^Federal Medical Biological Agency, Moscow, Russia

^21^Cardiovascular & Metabolic Disorders Program, and Center for Computational Biology, Duke-NUS Graduate Medical School, Singapore

^22^Kuopio Research Institute of Exercise Medicine, University of Eastern Finland, Kuopio, Finland

^23^ School of Public Health, University of South Carolina, Columbia, SC, USA

* These authors contributed equally

**Table of Contents**

**Supporting Information**

1. **Additional Information on the Contributing Studies**

**S1 Table. The Discovery Phase: The GENATHLETE Cohort.**

**S1 Fig. Distribution of GENATHLETE VO_2_max of the 315 Elite Endurance Athletes.**

**S2 Fig. Quantile-Quantile plot of observed vs expected –log 10 p values for genome-wide data from Japanese athletes and controls.**

1. **Regional Association Plots for the Japanese Athletes and Controls**

**S3 Fig. Regional association plot of the index SNP – rs921665.**

**S4 Fig. Regional association plot of the index SNP – rs6548153.**

**S5 Fig. Regional association plot of the index SNP – rs7650685.**

**S6 Fig. Regional association plot of the index SNP – rs10007111.**

**S7 Fig. Regional association plot of the index SNP – rs558129.**

**S8 Fig. Regional association plot of the index SNP – rs2910756.**

**S9 Fig. Regional association plot of the index SNP – rs11975386.**

**S10 Fig. Regional association plot of the index SNP – rs16906888.**

**S11 Fig. Regional association plot of the index SNP – rs17690338.**

**S12 Fig. Regional association plot of the index SNP – rs2761291.**

**S13 Fig. Regional association plot of the index SNP – rs4541108.**

**S14 Fig. Regional association plot of the index SNP – rs10874242.**

**S15 Fig. Regional association plot of the index SNP – rs12047209.**

**S16 Fig. Regional association plot of the index SNP – rs2361506.**

**S17 Fig. Regional association plot of the index SNP – rs9355947.**

**S18 Fig. Regional association plot of the index SNP – rs6959675.**

**S19 Fig. Regional association plot of the index SNP – rs3780169.**

**S20 Fig. Regional association plot of the index SNP – rs9580890.**

**S21 Fig. Regional association plot of the index SNP – rs2694093.**

**A. Additional information on the contributing studies**

**GENATHLETE**

Even though the minimal VO_2_max level required to be part of the GENATHLETE study was 75 ml O_2_/kg/min, there was a range of values among the 315 endurance athletes. Supplementary Table S1 presents the basic description of the athletes and controls of GENEATHLETE. All GENATHLETE participants were males. Cases and controls were matched on ethnicity and country of origin. The controls were sedentary individuals with a low maximal aerobic capacity.

**S1 Table. The Discovery Phase: The GENATHLETE Cohort**

**(mean**± **SD)**

|  | **Endurance Athletes** | **Sedentary Controls** |
| --- | --- | --- |
| N | 315 | 320 |
| Height (cm) | 178.7±6.1 | 178.1±7.3 |
| Weight (kg) | 69.2±7.1 | 76.5±11.2 |
| VO_2_max (ml/kg/min) | 79.0±3.4 | 40.0±7.1 |
| Country of origin [n (%)]: |  |  |
| - Germany | 187 (59.2) | 173 (54.1) |
| - USA/Canada | 78 (25.0) | 101 (31.6) |
| - Finland | 50 (15.8) | 46 (14.3) |

S1 Fig shows the distribution across three classes of VO_2_max levels. About 7% of these athletes had a maximal oxygen uptake greater than 85 ml O_2_/kg/min.


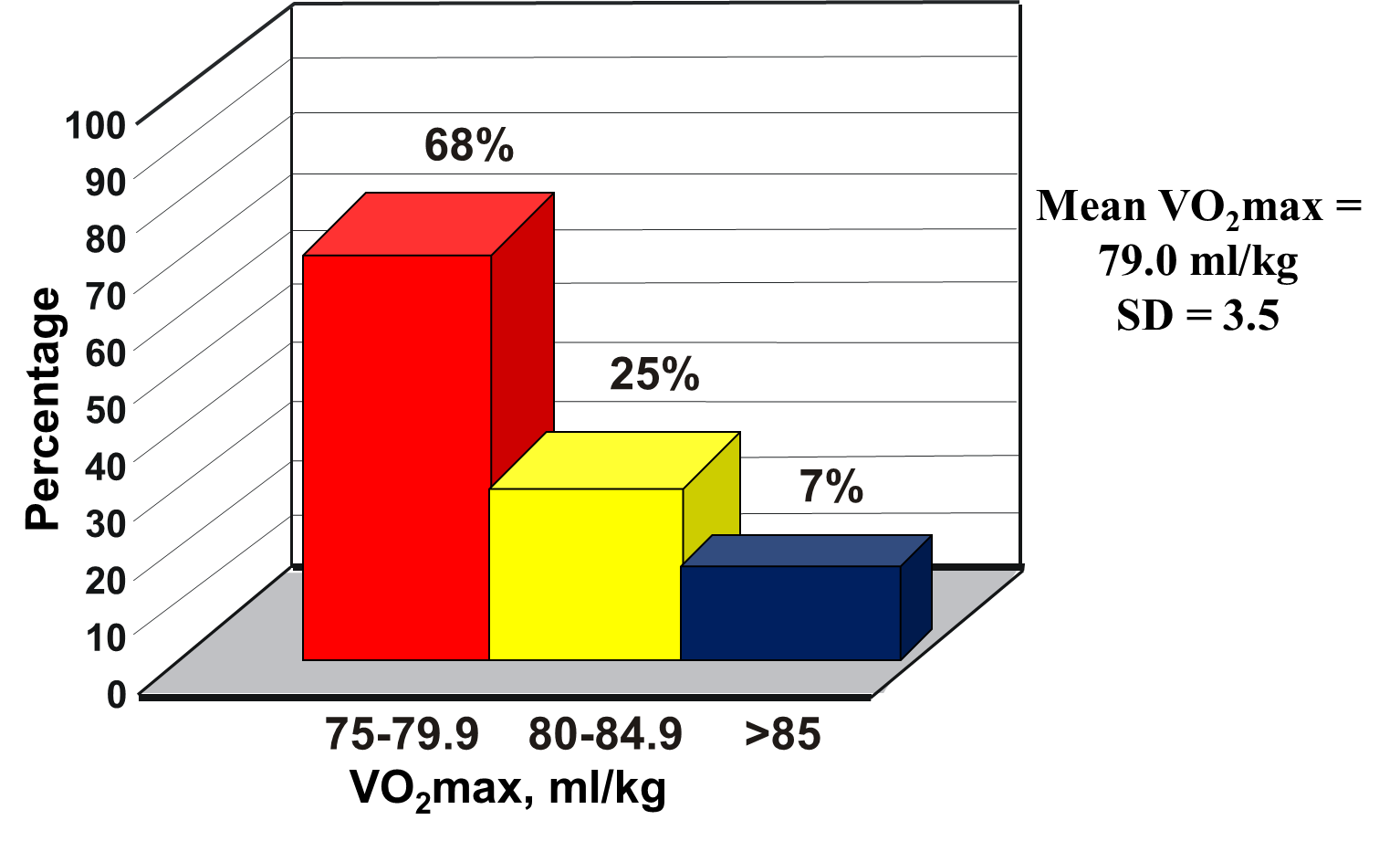


**S1 Fig. Distribution of GENATHLETE VO_2_max of the 315 Elite Endurance Athletes**

In GENATHLETE, genomic DNA was extracted from whole-blood samples by commercial DNA extraction kits (Gentra Systems, Inc., Minneapolis, MN), and the DNA stock samples were diluted to 50 ng/µL concentrations. SNPs for the study were those captured in the Illumina CardioMetabochip (Illumina Inc., San Diego, CA), which contains over 195,000 genetic markers including ~66,000 variants implicated in the aetiology of cardiometabolic traits and disease outcomes from discovery GWAS cohorts, as well as variants around known loci for the purposes of fine-mapping[^1^](#_ENREF_1). The SNPs were genotyped using the Illumina Infinium II assay on Illumina iScan platform. All post-amplification steps of the assay protocol were automated with Tecan Freedom EVO liquid-handling robotics to assure uniform and efficient sample processing. The genotype calls were performed with the Illumina GenomeStudio software, and all samples were called in the same batch to eliminate batch-to-batch variation. All GenomeStudio genotype calls with a GenTrain score less than 0.90 were checked and confirmed manually.

For genotyping quality control purposes, four *Centre d’Etudes sur les Polymorphismes Humains* DNA samples (NA10851, NA10857, NA10860, NA10861) were genotyped along with the GENATHLETE samples. The same samples are included in the HapMap Caucasian reference cohort, which allows monitoring our genotyping performance against that of other laboratories. Concordance between our results with the HapMap data was 100%. SNPs showing marked deviation from Hardy-Weinberg equilibrium (HWE) (p < 0.00001) were excluded. However, since deviations from HWE may be related to case-control status-related differences of genotype frequencies, identical non-HWE pattern was confirmed both in endurance athletes and non-athlete controls before the SNP was excluded from the database. A total of 143,000 SNPs were polymorphic and passed the quality control filters.

**Japanese Cohort of Endurance Athletes and Controls**

**For GWAS in the discovery phase:**

The subjects consisted of 60 elite Japanese endurance runners and 118 healthy Japanese controls. All athletes were international athletes (participants in Olympic Games, World and Asian Championships) and the group included several medalists at these international games. Controls comprised of individuals who had never excelled in sport and recruited from healthy Japanese men and women living in Tokyo and the surrounding areas. The participants were informed of the purpose and methods used in the study and each provided written informed consent to participate. The study was approved by the ethics committee of Juntendo University, Tokyo Metropolitan Institute of Gerontology and National Institute of Health and Nutrition in Japan and was conducted in accordance with the Declaration of Helsinki for Human Research. Total DNA was isolated from saliva or venous blood by use of Oragene･DNA Collection Kits (DNA genotek, Ontario, Canada) or QIAamp DNA blood Maxi Kit (QIAGEN, Hilden, Germany), respectively. Total DNA samples were genotyped for more than 700,000 markers using the Illumina^®^ HumanOmniExpress Beadchip. The genotype calls were performed with the Illumina GenomeStudio software. PLINK[^2^](#_ENREF_2) (see also http://pngu.mgh.harvard.edu/~purcell/plink/contact.shtml#cite) was used for quality control checks and association analyses. Samples with discordant sex, genotype missing rate > 5%, autosomal heterozygosity rate > ±3 standard deviations from the mean heterozygosity, all pairs of samples with high identity-by-descent (IBD) sharing > 0.05, and apparent outliers following principal component analysis were removed. Markers with call rate < 95% were excluded. Likewise, markers showing significant deviation from Hardy-Weinberg Equilibrium (*P* ≤ 0.0000001) were dropped, as were variants with a minor allele frequency (MAF) < 0.01. Ten principal components were extracted using EIGENSOFT package[^3^](#_ENREF_3)^,^[^4^](#_ENREF_4) to examine marker allele frequency variation between the Japanese samples and five HapMap reference populations and to explore/correct population stratification and other subtle differences due to assay/laboratory effects. After removing SNPs and individuals failing quality control, 541,179 autosomal SNPs in 60 Japanese endurance athletes and 116 Japanese controls were available for association analyses.

Regional association plots of the top signals crossing unadjusted *P* < 5x10^-5^ were also created using LocusZoom Version 1.1[^5^](#_ENREF_5) (http://csg.sph.umich.edu/locuszoom), including information on the location of genes and levels of linkage disequilibrium (LD). Each individual plot was specified by the SNP of interest, treated as the key marker for that region. A 500 kb flanking region on each side of the index SNP was specified. Plots were generated based on Human Genome19 (hg19). LD levels between the index SNP and the surrounding SNPs, as well as putative recombination rates, were calculated by LocusZoom using 1000G Mar 2012 ASN as the reference population. By examining the regional association plots, putative genomic regions were identified after discarding redundant signals.

**Additional Japanese samples for the meta-analysis phase:**

The additional subjects for the replication phase consisted of 143 sub-elite Japanese endurance runners and 692 healthy Japanese controls. All athletes were national athletes (participants in national competition of Japan). Controls comprised of individuals who had never excelled in sport and recruited from healthy Japanese men and women living in Tokyo and the surrounding areas. The participants were informed of the purpose and methods of the study and each provided written informed consent to participate. The study was approved by the ethics committee of Juntendo University, Tokyo Metropolitan Institute of Gerontology and National Institute of Health and Nutrition in Japan and was conducted in accordance with the Declaration of Helsinki for Human Research. Total DNA was isolated from saliva or venous blood by use of Oragene･DNA Collection Kits (DNA genotek, Ontario, Canada) or QIAamp DNA blood Maxi Kit (QIAGEN, Hilden, Germany), respectively.

**Study of Athletes from Australia**

The nonathlete (control, N=167) population consists of unrelated white Australians from three different sources (blood donors, healthy children participating in an unrelated study, healthy adults participating in a talent-identification program with the Australian Institute of Sport). All athletes are Caucasian Australian (n = 215). Athletes were classified as elite if they had represented Australia in their sport at an international level (n = 167; 39 of these athletes competed in an Olympic Games). Elite athletes competed in a number of different endurance events, including athletics (>800 m n = 20), cycling (road n = 46 and track n = 24), rowing (n = 72), and long-distance swimming (n = 5). The sub-elite athlete population (n = 48) includes triathletes and multi-sport eventers (n = 28), surf life-saving (n = 13), and cross-country skiing (n = 7). This study was approved by the institutional review boards of the Children’s Hospital at Westmead, the University of Sydney, and the Australian Institute of Sport.

The 45 SNPs targeted for replication were genotyped with the Illumina GoldenGate assay with VeraCode beads and the BeadXpress platform for the samples from Japan, Ethiopia, Kenya, and Spain. The same panel of SNPs was genotyped with Sequenom MassARRAY platform for the athletes and controls from Australia and Poland. Genomic DNA from both the Australian and Polish cohorts (athletes and controls) was whole genome amplified (WGA) using the Illustra GenomiPhi V2 DNA amplification Kit (GE Healthcare, NSW, Australia). Prior to Sequenom analysis, WGA DNA was assessed using the Quant-iT PicoGreen dsDNA assay kit (Life Technologies, VIC, Australia). Sequenom MassARRAY was performed by the Australian Genome Research Facility (AGRF, Brisbane, Australia) using a minimum of 20ng of amplified DNA.

**Study of Athletes from Poland**

The procedures followed in the study were conducted according to the principles of the World Medical Association Declaration of Helsinki. The procedures followed in the study were approved by the Pomeranian Medical University Ethics Committee (approval nr BN-001/45/08). All participants were given a consent form and a written information sheet concerning the study, providing all pertinent information (purpose, procedures, risks, benefits of participation). The potential participant had time to read the information sheet and the consent form. After ensuring that the participant has understood the information every participant gave written informed consent (signed consent form) to genotyping on the understanding that it was anonymous and that the obtained results would be confidential. The experimental procedures were conducted in accordance with the set of guiding principles for reporting the results of genetic association studies defined by the STrengthening the REporting of Genetic Association studies (STREGA) Statement.

**Study of Athletes from Spain**

All athletes (n= 170) are Spanish (Caucasian) men with solid expertise in international competitions. We collected blood/saliva samples over the last 4 years in different places of Spain from endurance athletes who were runners (mostly 5,000m and above), professional road cyclists, rowers, canoeists and triathletes. Inclusion criteria for this group were: having been finalist in ≥1 edition of Olympiads or World/European championships (in either ‘absolute’ or under-23-years (‘sub23’) or ‘junior’ category), except for cyclists. For the latter, having won ≥1 stage of the major 3week races (Vuelta, Giro or Tour) sufficed as inclusion criteria.

We also collected saliva samples from control, disease-free subjects (n=198) who were also all Spanish (Caucasian) men, either sedentary undergraduate students living in Madrid or recreational runners.

All subjects provided written consent and the study protocol was approved by the Ethics committee of Universidad Pablo Olavide. DNA sampling was done in Madrid (researchers TY, CS)

To assign athletes (all but cyclists) to the upper or lower 50% in terms of performance, we 1st established the following ranking system based on 6 categories (ranging from higher lo lower performance):

1             1st to 3rd in Olympic Games

2             1st to 3rd in Word Championships

3             4th to 8th in Olympic Games

4             1st to 3rd in European Championships

5             4th to 8th in World Championships

6             4th-8th in European Championships

As for cyclists, the 50% top ones were those able to finish a major 3wk race in top-3 position. The final number of subjects with acceptable genotype calls ranged from 165 to 170 in athletes and from 184 to 198 in controls.

**Study of Athletes from Russia**

The study involved 153 Russian athletes (86 males and 67 females) endurance athletes (biathletes (n=28), cross-country skiers (n=36), race walkers (n=8), 5-25 km swimmers (n=14), marathon runners (n=2), rowers (n=33), 3-10 km runners (n=14), 800-1500 m swimmers (n=7), triathletes (n=8), 5-10 km speed skaters (n=3). There were 68 athletes classified as ‘elite’ (prize winners of major international competitions). The other athletes (n = 85) were classified as ‘sub-elite’ athletes (participants in international competitions). Controls were 841 (533 males and 308 females) healthy, unrelated citizens of Russia. The athletes and controls were all Caucasians of Eastern European descent.

**Genome-wide genotyping**

Four ml of venous blood of all Russian athletes (n=153) and 192 Russian controls were collected in tubes containing EDTA (Vacuette EDTA tubes, Greiner Bio-One, Austria). Blood samples were transported to the laboratory at 4°C and DNA was extracted on the same day. DNA extraction and purification were performed using a commercial kit according to the manufacturer's instructions (Technoclon, Russia) and included chemical lysis, selective DNA binding on silica spin columns and ethanol washing. Extracted DNA quality was assessed by agarose gel electrophoresis at this step. HumanOmni1-Quad BeadChips (Illumina Inc, USA) were used for genotyping and 37 of the replication SNPs (rs10874242, rs12047209, rs921665, rs2694093, rs13032068, rs2361506, rs7650685, rs10938202, rs10007111, rs4699824, rs558129, rs2910756, rs611601, rs9355947, rs11975386, rs17055965, rs16906888, rs7861665, rs3780169, rs17690338, rs7947391, rs1815739, rs5443, rs10859809, rs9583073, rs214018, rs10220831, rs4777189, rs4288991, rs8065364, rs3745349, rs12460848, rs6548153, rs2761291, rs4541108, rs6959675, rs9580890) were included in the BeadChip. The assay required 200 ng of DNA sample as input with a concentration of at least 50 ng/µl. Exact concentrations of DNA in each sample were measured using a Qubit Fluorometer (Invitrogen, USA). All further procedures were performed according to the Infinium HD Assay instructions.

**Genotyping with RT-PCR**

Genomic DNA of 649 Russian controls was isolated from leukocytes in venous blood by proteinase K (Serva) digestion followed by phenol/chloroform extraction and ethanol precipitation. Genotyping of 16 replication SNPs (rs12047209, rs2694093, rs13032068, rs558129, rs2910756, rs17055965, rs7861665, rs1815739, rs5443, rs10859809, rs10220831, rs4777189, rs4288991, rs8065364, rs3745349, rs6548153) was carried out with TaqMan probes at iQ5 PCR Thermal Cycler (Bio-Rad). PCR was performed in 25 μl reaction volumes containing 20–100 ng of genomic DNA, 65 mMTris-HCl (рН 8.9), 23 mM ammonium sulphate, 3 mM MgCl2, 0.05% Tween 20, 0.2 mMdNTP, 0.3 μM of each primer, 0.1 μM of each probe and 1.0 U of Taq polymerase. PCR thermal cycling conditions were as follows: denaturation for 2 min at 95°С followed by 49 cycles each for 8 s at 95°С and 40 s at 58°С. dNTP, TaqMan probes, oligonucleotide primers and Taq polymerase were synthesized by the Institute of Chemical Biology and Fundamental Medicine (Siberian Branch, Russian Academy of Sciences).

**Study of Athletes from Ethiopia**

The Ethiopian sample set is comprised of 75 elite Ethiopian endurance athletes and 198 controls (89 Arsi controls and 109 controls from the general population). 75 athletes (participating in 5km, 10km or Marathon) were from the Ethiopian junior- and senior-level national teams. These athletes had finished in a top-3 position in national trails and met the International Association of Athletics Federations "A" standard for entry[^6^](#_ENREF_6). They had represented Ethiopia in international competitions, including World Championships and/or Olympic Games. They are considered as elite athletes. Given greater representation of Arsi population in Ethiopian endurance athletes [^7^](#_ENREF_7), an additional 89 Arsi controls were recruited along with another 109 controls representing the general Ethiopian population to best geographically match athletes to controls. Written informed consents were obtained from all participants and ethical approvals were obtained from the Oxford Tropical Research Ethics Committee, the University of Glasgow Ethics Committee for Non-Clinical Research Involving Human Subjects, and a committee from the Ethiopian Athletics Federation.

Buccal cell samples were collected from each subject and stored in cell lysis buffer (0.1 M Tris-HCl pH 8.0, 0.1 M EDTA, 1% SDS). DNA was extracted using the QIAamp^®^ DNA Mini kit (QIAgen, Hilden, Germany) according to the instructions of the manufacturer with minor adjustments. Following extraction, DNA samples were quantified using a Nanodrop® ND-8000 spectrophotometer (Nanodrop Technologies, Wilmington, DE, USA). Working DNA samples were stored at 4°C during the genotyping analysis.

**Study of Athletes from Kenya**

278 Kenyan endurance athletes (distances ranging from 3km to marathon) and 85 controls subjects were sampled. 66 of the athletes had represented Kenya in international competitions, including many record holders, Olympic, World and Commonwealth champions. The remaining 212 athletes had competed at national level in Kenya. Controls were students at Kenyatta University and were representative of the general Kenyan popluation[^7^](#_ENREF_7).Written informed consent was obtained from all participants, and ethical approval was obtained from the University Ethics Committee and local authorities in Kenya. Buccal cells (stored in cell lysis buffer, containing 0.1 M Tris-HCl pH 8.0, 0.1 M EDTA, 1% SDS) were collected from all Kenyan subjects. DNA isolation and quantification were carried out as previously described above for the Ethiopian cohort.

**Literature cited**

1. Voight BF, Kang HM, Ding J, et al. The metabochip, a custom genotyping array for genetic studies of metabolic, cardiovascular, and anthropometric traits. *PLoS Genet.* 2012;8(8):e1002793.

2. Purcell S, Neale B, Todd-Brown K, et al. PLINK: a tool set for whole-genome association and population-based linkage analyses. *Am J Hum Genet.* Sep 2007;81(3):559-575.

3. Price AL, Patterson NJ, Plenge RM, Weinblatt ME, Shadick NA, Reich D. Principal components analysis corrects for stratification in genome-wide association studies. *Nat Genet.* Aug 2006;38(8):904-909.

4. Patterson N, Price AL, Reich D. Population structure and eigenanalysis. *PLoS Genet.* Dec 2006;2(12):e190.

5. Pruim RJ, Welch RP, Sanna S, et al. LocusZoom: regional visualization of genome-wide association scan results. *Bioinformatics.* Sep 15 2010;26(18):2336-2337.

6. Ash GI, Scott RA, Deason M, et al. No association between ACE gene variation and endurance athlete status in Ethiopians. *Medicine and science in sports and exercise.* Apr 2011;43(4):590-597.

7. Scott RA, Georgiades E, Wilson RH, Goodwin WH, Wolde B, Pitsiladis YP. Demographic characteristics of elite Ethiopian endurance runners. *Medicine and science in sports and exercise.* Oct 2003;35(10):1727-1732.

1. **Regional Association Plots for the Japanese Athletes and Controls**

**S3 Fig. Regional association plot of the index SNP – rs921665.**

**S4 Fig. Regional association plot of the index SNP – rs6548153.**

**S5 Fig. Regional association plot of the index SNP – rs7650685.**

**S6 Fig. Regional association plot of the index SNP – rs10007111.**

**S7 Fig. Regional association plot of the index SNP – rs558129.**

**S8 Fig. Regional association plot of the index SNP – rs2910756.**

**S9 Fig. Regional association plot of the index SNP – rs11975386.**

**S10 Fig. Regional association plot of the index SNP – rs16906888.**

**S11 Fig. Regional association plot of the index SNP – rs17690338.**

**S12 Fig. Regional association plot of the index SNP – rs2761291.**

**S13 Fig. Regional association plot of the index SNP – rs4541108.**

**S14 Fig. Regional association plot of the index SNP – rs10874242.**

**S15 Fig. Regional association plot of the index SNP – rs12047209.**

**S16 Fig. Regional association plot of the index SNP – rs2361506.**

**S17 Fig. Regional association plot of the index SNP – rs9355947.**

**S18 Fig. Regional association plot of the index SNP – rs6959675.**

**S19 Fig. Regional association plot of the index SNP – rs3780169.**

**S20 Fig. Regional association plot of the index SNP – rs9580890.**

**S21 Fig. Regional association plot of the index SNP – rs2694093.**
